# GA4GH: International policies and standards for data sharing across genomic research and healthcare

**DOI:** 10.1016/j.xgen.2021.100029

**Published:** 2021-11-10

**Authors:** Heidi L. Rehm, Angela J.H. Page, Lindsay Smith, Jeremy B. Adams, Gil Alterovitz, Lawrence J. Babb, Maxmillian P. Barkley, Michael Baudis, Michael J.S. Beauvais, Tim Beck, Jacques S. Beckmann, Sergi Beltran, David Bernick, Alexander Bernier, James K. Bonfield, Tiffany F. Boughtwood, Guillaume Bourque, Sarion R. Bowers, Anthony J. Brookes, Michael Brudno, Matthew H. Brush, David Bujold, Tony Burdett, Orion J. Buske, Moran N. Cabili, Daniel L. Cameron, Robert J. Carroll, Esmeralda Casas-Silva, Debyani Chakravarty, Bimal P. Chaudhari, Shu Hui Chen, J. Michael Cherry, Justina Chung, Melissa Cline, Hayley L. Clissold, Robert M. Cook-Deegan, Mélanie Courtot, Fiona Cunningham, Miro Cupak, Robert M. Davies, Danielle Denisko, Megan J. Doerr, Lena I. Dolman, Edward S. Dove, L. Jonathan Dursi, Stephanie O.M. Dyke, James A. Eddy, Karen Eilbeck, Kyle P. Ellrott, Susan Fairley, Khalid A. Fakhro, Helen V. Firth, Michael S. Fitzsimons, Marc Fiume, Paul Flicek, Ian M. Fore, Mallory A. Freeberg, Robert R. Freimuth, Lauren A. Fromont, Jonathan Fuerth, Clara L. Gaff, Weiniu Gan, Elena M. Ghanaim, David Glazer, Robert C. Green, Malachi Griffith, Obi L. Griffith, Robert L. Grossman, Tudor Groza, Jaime M. Guidry Auvil, Roderic Guigó, Dipayan Gupta, Melissa A. Haendel, Ada Hamosh, David P. Hansen, Reece K. Hart, Dean Mitchell Hartley, David Haussler, Rachele M. Hendricks-Sturrup, Calvin W.L. Ho, Ashley E. Hobb, Michael M. Hoffman, Oliver M. Hofmann, Petr Holub, Jacob Shujui Hsu, Jean-Pierre Hubaux, Sarah E. Hunt, Ammar Husami, Julius O. Jacobsen, Saumya S. Jamuar, Elizabeth L. Janes, Francis Jeanson, Aina Jené, Amber L. Johns, Yann Joly, Steven J.M. Jones, Alexander Kanitz, Kazuto Kato, Thomas M. Keane, Kristina Kekesi-Lafrance, Jerome Kelleher, Giselle Kerry, Seik-Soon Khor, Bartha M. Knoppers, Melissa A. Konopko, Kenjiro Kosaki, Martin Kuba, Jonathan Lawson, Rasko Leinonen, Stephanie Li, Michael F. Lin, Mikael Linden, Xianglin Liu, Isuru Udara Liyanage, Javier Lopez, Anneke M. Lucassen, Michael Lukowski, Alice L. Mann, John Marshall, Michele Mattioni, Alejandro Metke-Jimenez, Anna Middleton, Richard J. Milne, Fruzsina Molnár-Gábor, Nicola Mulder, Monica C. Munoz-Torres, Rishi Nag, Hidewaki Nakagawa, Jamal Nasir, Arcadi Navarro, Tristan H. Nelson, Ania Niewielska, Amy Nisselle, Jeffrey Niu, Tommi H. Nyrönen, Brian D. O’Connor, Sabine Oesterle, Soichi Ogishima, Vivian Ota Wang, Laura A.D. Paglione, Emilio Palumbo, Helen E. Parkinson, Anthony A. Philippakis, Angel D. Pizarro, Andreas Prlic, Jordi Rambla, Augusto Rendon, Renee A. Rider, Peter N. Robinson, Kurt W. Rodarmer, Laura Lyman Rodriguez, Alan F. Rubin, Manuel Rueda, Gregory A. Rushton, Rosalyn S. Ryan, Gary I. Saunders, Helen Schuilenburg, Torsten Schwede, Serena Scollen, Alexander Senf, Nathan C. Sheffield, Neerjah Skantharajah, Albert V. Smith, Heidi J. Sofia, Dylan Spalding, Amanda B. Spurdle, Zornitza Stark, Lincoln D. Stein, Makoto Suematsu, Patrick Tan, Jonathan A. Tedds, Alastair A. Thomson, Adrian Thorogood, Timothy L. Tickle, Katsushi Tokunaga, Juha Törnroos, David Torrents, Sean Upchurch, Alfonso Valencia, Roman Valls Guimera, Jessica Vamathevan, Susheel Varma, Danya F. Vears, Coby Viner, Craig Voisin, Alex H. Wagner, Susan E. Wallace, Brian P. Walsh, Marc S. Williams, Eva C. Winkler, Barbara J. Wold, Grant M. Wood, J. Patrick Woolley, Chisato Yamasaki, Andrew D. Yates, Christina K. Yung, Lyndon J. Zass, Ksenia Zaytseva, Junjun Zhang, Peter Goodhand, Kathryn North, Ewan Birney

**Affiliations:** 1 Broad Institute of MIT and Harvard, Cambridge, MA, USA; 2 Massachusetts General Hospital, Boston, MA, USA; 3 Global Alliance for Genomics and Health, Toronto, ON, Canada; 4 Ontario Institute for Cancer Research, Toronto, ON, Canada; 5 Brigham and Women’s Hospital, Boston, MA, USA; 6 DNAstack, Toronto, ON, Canada; 7 University of Zurich, Zurich, Switzerland; 8 SIB Swiss Institute of Bioinformatics, Lausanne, Switzerland; 9 McGill University, Montreal, QC, Canada; 10 University of Leicester, Leicester, UK; 11 University of Lausanne, Lausanne, Switzerland; 12 CNAG-CRG, Centre for Genomic Regulation (CRG), The Barcelona Institute of Science and Technology, Barcelona, Spain; 13 Universitat Pompeu Fabra (UPF), Barcelona, Spain; 14 Universitat de Barcelona, Barcelona, Spain; 15 Wellcome Sanger Institute, Hinxton, UK; 16 Australian Genomics, Parkville, VIC, Australia; 17 Murdoch Children’s Research Institute, Parkville, VIC, Australia; 18 Canadian Center for Computational Genomics, Montreal, QC, Canada; 19 University of Toronto, Toronto, ON, Canada; 20 University Health Network, Toronto, ON, Canada; 21 Vector Institute, Toronto, ON, Canada; 22 Oregon Health and Science University, Portland, OR, USA; 23 European Molecular Biology Laboratory, European Bioinformatics Institute (EMBL-EBI), Hinxton, UK; 24 PhenoTips, Toronto, ON, Canada; 25 Walter and Eliza Hall Institute of Medical Research, Parkville, VIC, Australia; 26 University of Melbourne, Melbourne, VIC, Australia; 27 Vanderbilt University Medical Center, Nashville, TN, USA; 28 National Cancer Institute, National Institutes of Health, Bethesda, MD, USA; 29 Memorial Sloan Kettering Cancer Center, New York, NY, USA; 30 Nationwide Children’s Hospital, Columbus, OH, USA; 31 The Ohio State University, Columbus, OH, USA; 32 National Heart, Lung, and Blood Institute, National Institutes of Health, Bethesda, MD, USA; 33 Stanford University, Stanford, CA, USA; 34 UC Santa Cruz Genomics Institute, Santa Cruz, CA, USA; 35 Arizona State University, Washington, DC, USA; 36 Sage Bionetworks, Seattle, WA, USA; 37 University of Edinburgh, Edinburgh, UK; 38 Canadian Distributed Infrastructure for Genomics (CanDIG), Toronto, ON, Canada; 39 University of Utah, Salt Lake City, UT, USA; 40 Sidra Medicine, Doha, Qatar; 41 Weill Cornell Medicine - Qatar, Doha, Qatar; 42 Addenbrooke’s Hospital, Cambridge, UK; 43 University of Chicago, Chicago, IL, USA; 44 Mayo Clinic, Rochester, MN, USA; 45 National Human Genome Research Institute, National Institutes of Health, Bethesda, MD, USA; 46 Verily Life Sciences, South San Francisco, CA, USA; 47 Harvard Medical School, Boston, MA, USA; 48 Washington University School of Medicine in St. Louis, St. Louis, MO, USA; 49 Pryzm Health, Sydney, QLD, Australia; 50 Centre for Genomic Regulation (CRG), The Barcelona Institute of Science and Technology, Barcelona, Spain; 51 University of Colorado Anschutz Medical Campus, Aurora, CO, USA; 52 Johns Hopkins University, Baltimore, MD, USA; 53 Autism Speaks, Princeton, NJ, USA; 54 Duke-Margolis Center for Health Policy, Washington, DC, USA; 55 The University of Hong Kong, Hong Kong, Hong Kong; 56 BBMRI-ERIC, Graz, Austria; 57 Masaryk University, Brno, Czech Republic; 58 National Taiwan University, Taipei City, Taiwan; 59 École polytechnique fédérale de Lausanne (EPFL), Lausanne, Switzerland; 60 Cincinnati Children’s Hospital Medical Center, Cincinnati, OH, USA; 61 Queen Mary University of London, London, UK; 62 SingHealth Duke-NUS Genomic Medicine Centre, Singapore, Republic of Singapore; 63 SingHealth Duke-NUS Institute of Precision Medicine, Singapore, Republic of Singapore; 64 University of Waterloo, Waterloo, ON, Canada; 65 Garvan Institute of Medical Research, Darlinghurst, NSW, Australia; 66 University of Glasgow, Glasgow, UK; 67 Canada’s Michael Smith Genome Sciences Centre, BC Cancer, Vancouver, BC, Canada; 68 University of Basel, Basel, Switzerland; 69 Osaka University, Suita, Japan; 70 University of Nottingham, Nottingham, UK; 71 University of Oxford, Oxford, UK; 72 National Center for Global Health and Medicine Hospital, Tokyo, Japan; 73 University of Tokyo, Tokyo, Japan; 74 ELIXIR Hub, Hinxton, UK; 75 Keio University School of Medicine, Tokyo, Japan; 76 mlin.net LLC, San Jose, CA, USA; 77 CSC–IT Center for Science, Espoo, Finland; 78 ELIXIR Finland, Espoo, Finland; 79 Faculty of Medicine, University Southampton, Southampton, UK; 80 Seven Bridges, Boston, MA, USA; 81 The Australian e-Health Research Centre, CSIRO, Herston, QLD, Australia; 82 Wellcome Connecting Science, Hinxton, UK; 83 University of Cambridge, Cambridge, UK; 84 Heidelberg Academy of Sciences and Humanities, Heidelberg, Germany; 85 H3ABioNet, Computational Biology Division, IDM, Faculty of Health Sciences, Cape Town, South Africa; 86 Japan Agency for Medical Research & Development (AMED), Tokyo, Japan; 87 RIKEN Center for Integrative Medical Sciences, Yokohama, Japan; 88 University of Northampton, Northampton, UK; 89 Institute of Evolutionary Biology (UPF-CSIC), Universitat Pompeu Fabra (UPF), Barcelona, Spain; 90 Institucio Catalana de Recerca i Estudis Avançats, Barcelona, Spain; 91 Barcelonabeta Brain Research Center (BBRC), Pasqual Maragall Foundation, Barcelona, Spain; 92 Genomic Medicine Institute, Geisinger, Danville, PA, USA; 93 Human Genetics Society of Australasia Education, Ethics & Social Issues Committee, Alexandria, NSW, Australia; 94 Tohoku University, Sendai, Japan; 95 Spherical Cow Group, New York, NY, USA; 96 Laura Paglione LLC, New York, NY, USA; 97 Amazon Web Services, Inc., Seattle, WA, USA; 98 Invitae, San Francisco, CA, USA; 99 Genomics England, London, UK; 100 The Jackson Laboratory, Farmington, CT, USA; 101 University of Connecticut, Farmington, CT, USA; 102 National Center for Biotechnology Information, National Library of Medicine, National Institutes of Health, Bethesda, MD, USA; 103 Patient-Centered Outcomes Research Institute (PCORI), Washington, DC, USA; 104 Bicgen Foundation Inc, Arlington Heights, IL, USA; 105 Congenica Ltd., Cambridge, UK; 106 University of Virginia, Charlottesville, VA, USA; 107 University of Michigan, Ann Arbor, MI, USA; 108 QIMR Berghofer Medical Research Institute, Herston, QLD, Australia; 109 Precision Health Research Singapore, Singapore, Republic of Singapore; 110 Genome Institute of Singapore, Singapore, Republic of Singapore; 111 University of Luxembourg, Esch-sur-Alzette, Luxembourg; 112 National Center for Global Health and Medicine, Tokyo, Japan; 113 California Institute of Technology, Pasadena, CA, USA; 114 Barcelona Supercomputing Center, Barcelona, Spain; 115 Health Data Research UK, London, UK; 116 Melbourne Law School, University of Melbourne, Parkville, VIC, Australia; 117 Google LLC, Kitchener, ON, Canada; 118 Section of Translational Medical Ethics, University Hospital Heidelberg, Heidelberg, Germany; 119 Indoc Research, Toronto, ON, Canada; 120 Canadian Centre for Computational Genomics, Montreal, QC, Canada; 121 European Molecular Biology Laboratory, Heidelberg, Germany; 122 MyOme, Inc, San Bruno, CA, USA; 123 Kelly Government Solutions, Rockville, MD, USA; 124 Datadex Inc., Toronto, ON, Canada; 125 Howard Hughes Medical Institute, University of California, Santa Cruz, CA, USA; 126 Salt Lake City, UT, USA

## Abstract

The Global Alliance for Genomics and Health (GA4GH) aims to accelerate biomedical advances by enabling the responsible sharing of clinical and genomic data through both harmonized data aggregation and federated approaches. The decreasing cost of genomic sequencing (along with other genome-wide molecular assays) and increasing evidence of its clinical utility will soon drive the generation of sequence data from tens of millions of humans, with increasing levels of diversity. In this perspective, we present the GA4GH strategies for addressing the major challenges of this data revolution. We describe the GA4GH organization, which is fueled by the development efforts of eight Work Streams and informed by the needs of 24 Driver Projects and other key stakeholders. We present the GA4GH suite of secure, interoperable technical standards and policy frameworks and review the current status of standards, their relevance to key domains of research and clinical care, and future plans of GA4GH. Broad international participation in building, adopting, and deploying GA4GH standards and frameworks will catalyze an unprecedented effort in data sharing that will be critical to advancing genomic medicine and ensuring that all populations can access its benefits.

## Introduction

The Universal Declaration of Human Rights states that everyone has the right to share in scientific advancement and its benefits.^1,2^ In order to fully deliver the benefits from genomic science to the broad human population, researchers and clinicians must come together to agree on common methods for collecting, storing, transferring, accessing, and analyzing molecular and other health-related data. Otherwise, this information will remain siloed within individual disease areas, institutions, countries, or other jurisdictions, locking away its potential to contribute to research and medical advances.

The Global Alliance for Genomics and Health (GA4GH) is a worldwide alliance of genomics researchers, data scientists, healthcare practitioners, and other stakeholders. We are collaborating to establish policy frameworks and technical standards for responsible, international sharing of genomic and other molecular data as well as related health data. Founded in 2013,^3^ the GA4GH community now consists of more than 1,000 individuals across more than 90 countries working together to enable broad sharing that transcends the boundaries of any single institution or country (see https://www.ga4gh.org).

In this perspective, we present the strategic goals of GA4GH and detail current strategies and operational approaches to enable responsible sharing of clinical and genomic data, through both harmonized data aggregation and federated approaches, to advance genomic medicine and research. We describe technical and policy development activities of the eight GA4GH Work Streams and implementation activities across 24 real-world genomic data initiatives (“Driver Projects”). We review how GA4GH is addressing the major areas in which genomics is currently deployed including rare disease, common disease, cancer, and infectious disease. Finally, we describe differences between genomic sequence data that are generated for research versus healthcare purposes, and define strategies for meeting the unique challenges of responsibly enabling access to data acquired in the clinical setting.

## Harnessing the Genomic Medicine Revolution

As the costs associated with human genomic sequencing continue to decline, genomic assays are increasingly used in both research and healthcare. As a result, we expect tens of millions of human whole-exome or whole-genome sequences to be generated within the next decade, with a high proportion of that data coming from the healthcare setting and therefore associated with clinical information.^4^ If they can be shared, these datasets hold great promise for research into the genetic basis of disease^5^ and will represent more diverse populations than have traditionally been accessible in research; however, data from individual healthcare systems are rarely accessible outside of institutional boundaries.

GA4GH aims to enable the responsible sharing of clinical and genomic data across both research and healthcare by developing standards and facilitating their uptake.^6^ We believe that without such a consortium, the emerging utility of genomics in clinical practice will be slower, more expensive, and fragmented, with little harmonization between countries.^7^ GA4GH standards (see Table 1) allow researchers to securely and responsibly access data regardless of where they are physically located. Technical standards give researchers the confidence that someone else could reproduce their work by running the same packaged method over the same underlying data, using the same persistent identifiers. Standards also give data providers confidence that their data are being accessed in accordance with their data use policies, by researchers they have authorized, without losing control of multiple downloaded copies of the data. As a result, data providers can enable research with the assurance that their legal and ethical requirements are being upheld, while researchers benefit from the use of global data resources and tools.

As nascent genomic medicine programs emerge in many countries, we believe that federated approaches (see Federated access below), in addition to centralized data sharing where feasible, are necessary to satisfy the goals of both the research and healthcare communities. In addition, many commercial and public organizations aim to minimize the costs and risks of the complex technical software needed to either contribute to genomic medicine or deliver genomic tools. A complex, multistakeholder ecosystem requires neutral and technically competent standards; these standards must be adaptable for disparate purposes and useful for the broad set of end-users: clinical, academic, commercial, and public. Finally, standards must be developed to intentionally support the global research community with specific attention to policies of equity, diversity, and inclusion to tangibly enable progress for all global communities.

## GA4GH Organization

**Figure 1:**
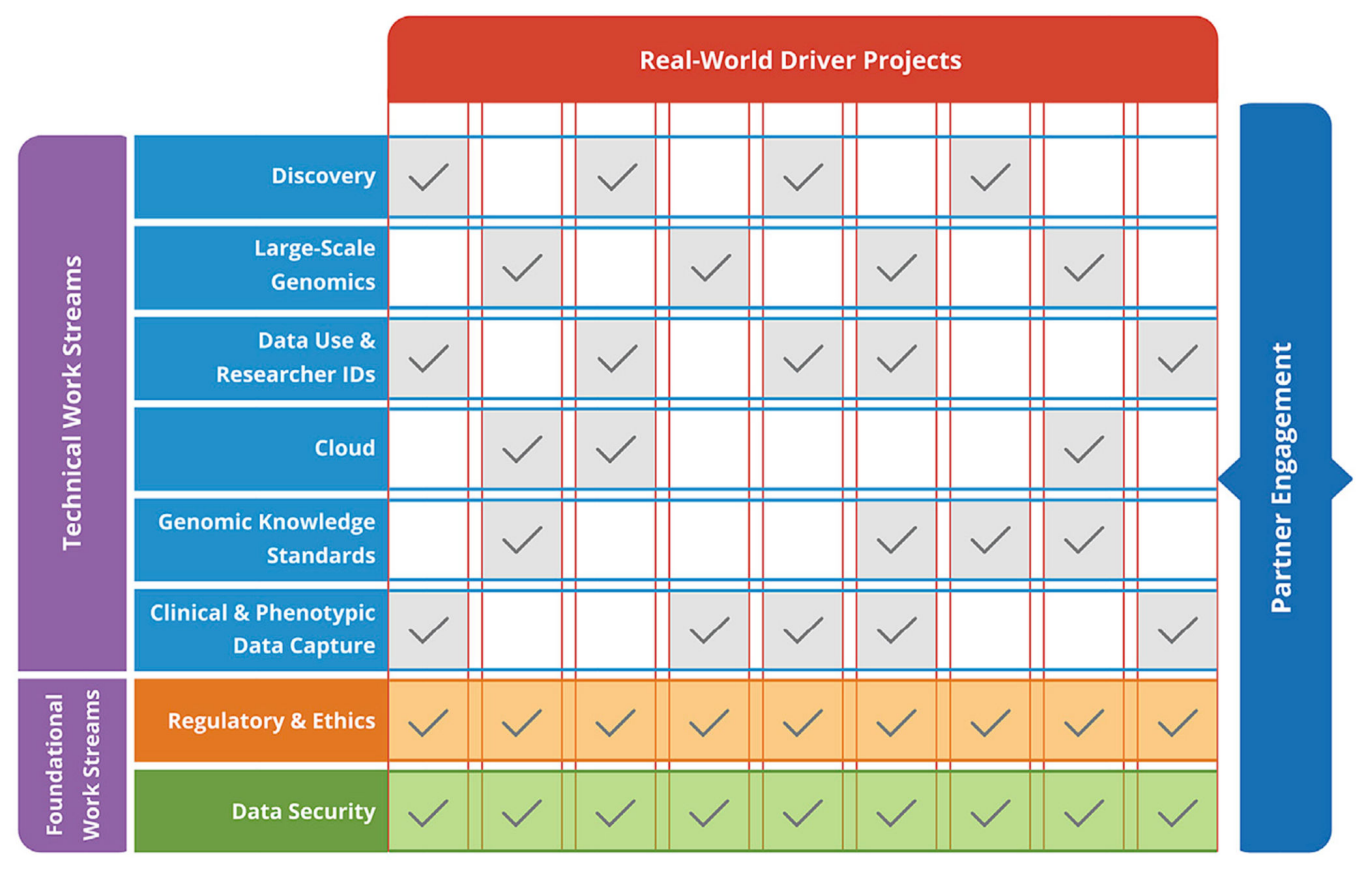
Matrix structure of the Global Alliance for Genomics and Health GA4GH is a community of diverse stakeholders from Driver Projects and other institutions working together in the context of Work Streams. Each GA4GH Driver Project is expected to dedicate two full-time equivalents across at least two GA4GH Work Streams. As foundational groups that review all GA4GH deliverables, the Regulatory and Ethics and Data Security Work Streams must have representation from every Driver Project. In addition to Driver Projects, any member of the community—regardless of domain, sector, nation, or affiliation—is invited to participate in any GA4GH Work Stream. Supplemental information includes details on how each of the 24 GA4GH Driver Projects intersects with the six technical Work Streams.

GA4GH has partnered with 24 real-world genomic data initiatives (Driver Projects) to ensure its standards are fit for purpose and driven by real-world needs. Driver Projects make a commitment to help guide GA4GH development efforts and pilot GA4GH standards (see Table 2). Each Driver Project is expected to dedicate at least two full-time equivalents to GA4GH standards development, which takes place in the context of GA4GH Work Streams (see Figure 1). Work Streams are the key production teams of GA4GH, tackling challenges in eight distinct areas across the data life cycle (see Box 1). Work Streams consist of experts from their respective sub-disciplines and include membership from Driver Projects as well as hundreds of other organizations across the international genomics and health community.

## GA4GH standards development and approval process

GA4GH Work Streams and Driver Projects have identified, and are actively developing, the technical specifications and policy frameworks they believe to be of most relevance to enable widespread data sharing, federated approaches, and interoperability across datasets to facilitate genomic research (see supplemental information for more details on the product development process); the areas of focus are outlined in Box 1, with individual products defined in Table 1 and in the 2020/2021 GA4GH Roadmap (https://www.ga4gh.org/roadmap).

Each GA4GH deliverable can be implemented on its own to enable interoperability and consistency in a single area. However, when implemented together, they support broader activities in the research and clinical domains and enable productive genomic data sharing and collaborative analyses that can leverage global datasets produced in distinct locations around the world.

Each approved GA4GH deliverable is reviewed by a panel of internal and external experts not involved in the product’s development, and then by the GA4GH Steering Committee (https://www.ga4gh.org/about-us/governance-and-leadership-2/#steering). GA4GH standards are not typically accredited by a national or international standards body, and instead follow a model inspired by the Internet Engineering Task Force (IETF; https://www.ietf.org) and the World Wide Web Consortium (W3C; http://www.w3.org). This enables a flexible and rapid response to community needs and a focus on lowering barriers to interoperability through the development and adoption of pragmatic standards. However, there are occasions when certain standards benefit from a more formal accreditation process, especially when there is a direct link into healthcare usage (see next section and Box 2).

### Alignment with other standards organizations

To achieve greater international coordination and consistency of standards development, GA4GH proactively collaborates with other standards development organizations working in genomics, e.g., Health Level Seven (HL7; http://www.hl7.org), International Organization for Standardization (ISO; https://www.iso.org), Open Biological and Biomedical Ontology Foundry (OBO; http://www.obofoundry.org/). While defined work processes between GA4GH and other standards development bodies are still under development, GA4GH has initiated several pilot projects to explore mechanisms of collaboration. One such approach is the submission of GA4GH standards to ISO’s technical committees for approval as ISO international standards. Using a product development timeline that aligns the ISO approval process with the GA4GH approval process, both communities are able to contribute to the development of a standard in a harmonized manner. These efforts expand the diversity of contributors to both organizations, leading to more robust and internationally applicable standards. Another approach, guided by HL7 working groups and experts, is the translation of GA4GH standards into HL7 Fast Health Interoperability Resources (FHIR) Implementation Guides. These implementation guides enable interoperability of GA4GH standards with clinical systems and accelerate the use of clinical data for research.

GA4GH also aims to support and interoperate with existing translational models, ontologies, and terminologies (e.g., FHIR, HGVS, OMOP, PCORnet, Human Phenotype Ontology, SNOMED CT) for clinical genetics and genomics.^21–23^ Before launching a new standards development project, GA4GH Work Streams are encouraged to complete a landscape analysis that both defines relevant existing standards and how they will influence the development of the new standard. Coordination activities—such as joint meetings, shared documentation, and process harmonization between GA4GH work and these health standards-focused efforts—are critical for bridging the research-clinical divide and keeping respective products aligned. This helps prevent unnecessary proliferation of redundant standards and minimizes the development of semantically and syntactically conflicting standards that could hamper large-scale interoperability and lead to confusion within the adopter community (see Box 2).

### Federated approaches

Federated approaches—the ability to analyze data across multiple distinct and secure sites—is increasingly seen as an important strategy where data cannot be pooled for legal or practical reasons. These approaches are characterized by independent organizations hosting data in secure processing environments (e.g., clouds, trusted research environments) while adopting technical standards that enable analysis at scale.^24^ Application programming interfaces (APIs) can be deployed to enable researchers and portable workflows to visit multiple databases even where the data and computing environment are variably configured.^25^ Tools like “identity federation” can facilitate even closer integration across organizations.^26–28,29^ GA4GH Driver Projects and other partners are beginning to implement cloud-based workflows built on GA4GH standards that allow scientists to share, access, and interrogate data stored at disparate sites around the globe. Some concrete examples of this access pattern include (1) the Data Coordination Platform of the Human Cell Atlas, an internationally federated compute environment for analyzing single-cell data; (2) Genomics England’s secure Research Environment for approved investigators to access the 100,000 Genomes Project dataset; (3) the NHGRI Genomic Data Science Analysis, Visualization, and Informatics Lab-space (AnVIL)^30^ and the Gen3 Data Commons, which provide cloud-based spaces for scientists to work with large-scale genomic and genomic-related datasets and shared tools; and (4) H3ABioNet, a bioinformatics platform that serves data from the Human Heredity and Health in Africa (H3Africa) network to researchers across the continent and provides containerized workflows for analysis of the data.

Because these workflows are built on interoperable standards, they allow for secure access and efficient discovery, portability, and analysis. With more instances like these, the global community will be able to harness the power of large data and improve the reach of genomic medicine research. The federation and transparency enabled by standards will also encourage greater willingness among non-western and other underrepresented populations to share their data, affording greater diversity in the overall data available and equity in its impacts.

## Genomics in Healthcare

The process of sequencing a genome is essentially the same in any setting, but the scale and quality control of production,^31^ as well as the regulation and dissemination of the resulting data, can be quite different in healthcare compared to research.^32,33^ “Research genomes” contain de-identified data and therefore are often openly shared with other researchers, including for funding and publishing requirements (for NIH policy, see web resources), frequently with managed access, e.g., via the European Genome-phenome Archive (EGA), the Japanese Genotype-phenotype Archive (JGA), or the database of Genotypes and Phenotypes (dbGaP). Researchers worldwide will draw on these openly shared genomic datasets for their own studies, increasing the amount of knowledge derived from each genome.^34^ However, while such research genomes are more readily available, these datasets usually do not include the type or extent of longitudinal, standardized, or interoperable clinical data needed for genomic medicine.^35^

Healthcare-based research and testing have an entirely different financial, legal, and social landscape, with the structure, provision, and regulation varying by country, covering the full spectrum from state-run to private schemes.^7^ In each system, the cost of an assay in healthcare—genomics included—is often considered in light of its benefits to the health of an individual and cost effectiveness within the healthcare system.^36^ In theory, if a genomic assay demonstrates clinical utility for a specific application within a healthcare system—especially if it is cost effective—the only limit to its deployment is the number of patients who will potentially benefit. In practice, however, there are logistical, financial, regulatory, educational, scientific, and clinical-based hurdles to overcome before a genomic test becomes a routine clinical offering. In addition, barriers to healthcare access will likely remain impediments to large-scale implementation in many countries.

The current case for implementing genomics in healthcare can be presented in four broad disease areas: rare disease, cancer, common/chronic disease, and infectious disease. In the following sections we outline the case for healthcare-funded sequencing in each disease area. We also highlight challenges to implementation in each area and GA4GH deliverables aimed at overcoming these issues.

### Rare disease

Arguably, the rare disease space has seen the most successful deployment of genomics in healthcare, with many reporting diagnostic rates of at least 20%–30%, and health economic studies demonstrating cost-effectiveness and diagnostic utility.^36–41^ Clinical geneticists have used single-gene or small gene panel tests since the early 1990s to support diagnosis and some treatment decisions for many of these diseases. The cost of assaying broader genomic regions—including exome and genome sequencing—has fallen considerably, with a substantial impact on rare-disease diagnosis and discovery research.^42,43^ However, with more than 10,000 rare diseases^44^ affecting more than 300 million patients worldwide^45^ diagnosing and discovering treatments for many of these diseases has been challenging. As such, the rare disease community has embraced data sharing in order to facilitate global knowledge exchange and improve patient diagnostic rates, understand disease progression, and augment care strategies.^41^

To further enable progress, clinical and research laboratories and health systems must support several key activities to effectively identify, diagnose, and eventually treat the genetic causes of rare disease: (1) aggregate genomic and phenotypic data, needed for discerning population allele frequencies in disease and non-disease populations and implicating new genes in rare disease; (2) catalog the validity of gene-disease associations using consistent annotation models and terminologies;^46^ (3) collectively build knowledge bases to understand variant pathogenicity; (4) define the natural histories of rare diseases to predict disease progression and enable a foundation upon which to develop clinical trials; and (5) monitor treatment efficacy of emerging therapeutics. GA4GH standards and policies already enable and will continue to build upon these activities. For example, the Matchmaker Exchange—a rare disease gene discovery platform which has benefited from GA4GH guidance on API-based data exchange formats as well as consent^47^ and data security policies^48,49^—illustrates the power of bringing practicing clinicians and researchers together, as cases from across the globe are necessary to build evidence to confirm new gene-disease relationships.^48^

GA4GH promotes knowledge sharing in ClinVar, a database which has accelerated improvements in variant classification across the clinical laboratory community.^50^ Additional methods are now being deployed to move beyond manual submission of variant classifications to a centralized database; such advances will enable more timely access to siloed laboratory knowledge and evidence-based variant classification. Realtime sharing with ClinVar—facilitated by APIs and with entries linked to rich, case-level data—will be needed to scale our understanding of the more than 750 million variants so far identified in the human genome (e.g., within gnomAD; https://gnomad.broadinstitute.org). The Variation Representation (VRS)^18^ and Variant Annotation (VA) specifications aim to support the exchange of variant data, Phenopackets and Pedigree representation to support the use of standardized clinical and family history data, as well as new APIs (e.g., Beacon v2 API and Data Connect API) to enable the identification of data for further access and analysis. The aim is for these standards to support a more global and federated approach to rare disease data and knowledge sharing that will be critical to advancing diagnosis and treatment of rare diseases.

### Cancer

One in five men and one in six women worldwide will have a cancer diagnosis in their lifetime.^51^ This risk is 2- to 3-fold greater in higher-resource countries,^51^ with estimates as high as one in two people in the UK for example.^52^ An altered somatic genome is a consistent hallmark of cancer, often associated with specific pathogenic mutations.^53^ In some individuals with hereditary cancer syndromes, germline variants can disrupt cancer-related pathways and increase the risk of developing a “heritable” malignancy.^54–56^ Characterizing a cancer by sequencing a patient’s tumor genome alongside their germline genome has resulted in profound insights into molecular mechanisms of malignant transformation and discovery of potential therapeutic targets.^57,58^ Tumor/normal sequencing has demonstrated applications in disease monitoring^59^ as well as diagnosis,^60^ prognosis,^61^ and therapeutic response prediction,^62^ both at initial presentation^63^ and disease recurrence.^64^

Applying cancer genomics in the clinic is more complicated than that for rare diseases. For cancer patients, treatment strategy time frames are commonly measured in weeks and incorporating genomic information within such an urgent turnaround time is logistically challenging to integrate into clinical decision making.^65^ Additionally, while the use of genomics for diagnosis and improved symptom management can lead to substantial improvements for rare disease patients and their families, application of genomics in cancer treatment is more complex and may include dual assessment of both somatic and germline genomes to determine heritable cancer risk and the assessment of the evolving tumor genome due to changing selective pressures in response to targeted therapies. Cancer genomic information is most useful if it informs treatment options, yet development of systems that match patients to appropriate clinical trials would be needed to fully realize the benefits of genomic tumor data where estimates of clinical trial enrollment in patients with cancer stands at ~8%.^66^ Genomic information is increasingly important in clinical decision making through routine clinical sequencing assays and molecular tumor boards.^67^ The heterogeneity of cancer as a disease—of each individual tumor and of any concurrent or subsequent manifestation, such as metastasis or recurrence—adds many layers of complexity to genomic analysis.^68^ To address this complexity, it is important to analyze somatic and germline variation data together to understand their contribution to cancer risk.^69^

Most of the same standards and workflows important for rare disease apply to tumor sequencing, including data storage and compression standards (e.g., CRAM), variation representation (e.g., VCF and VRS), analysis (e.g., cloud-based workflows), and linkage to patient records (e.g., Phenopackets). However, discovery of oncogenic driver mutations also requires significant coordination and standardization to track outcome data (e.g., progression and response to treatment), a key element in determining the clinical significance of variation found in cancer patients.^70^ As such, many groups have created knowledge bases to annotate cancer genomic variation associated with evidence of pathogenicity or relevant treatment options; however, these knowledge bases can have limited levels of interoperability. In 2014, a GA4GH task team launched the Variant Interpretation for Cancer Consortium (VICC), which standardizes and coordinates clinical somatic cancer curation efforts and has created an open community resource to provide the aggregated information.^71^ Moving forward, major oncogenomic resources are now working with GA4GH on the harmonization of variant interpretation evidence, through refinement and adoption of standards such as the Beacon API, the Data Use Ontology (DUO),^9^ VA, and VRS. Additionally, these standards are being implemented across multiple GA4GH Driver Projects (see Table 2) that capture genomic data and/or diagnostic variant interpretation across the longitudinal evolution of cancer.

### Common/chronic disease

“Common disease” is a catchall phrase describing a vast spectrum of diseases that have complex environmental and genetic etiologies. Accurate prediction of common diseases from genetics has been a topic of study since the inception of human genetics, yet genomic information is still not widely used in clinical practice for this purpose. The discovery of a large number of genetic susceptibility loci (polygenic architecture) supported the common-disease common-variant hypothesis^72^ and has led to the generation of polygenic risk scores summarizing common disease risk.^73^ Studies are now beginning to demonstrate the clinical benefits of applying polygenic risk scores in practice through stratification of the population for deploying disease management strategies.^74–76^ As the assay of choice moves from genotype arrays to sequencing, there will be integration between common disease and rare disease applications; this is already the case for certain diseases such as susceptibility to breast cancer^75^ or heart disease.^77^ When such genomic information can be used clinically for common diseases, it will be more justifiable to sequence entire populations. Population-scale sequencing is in place already in some countries (e.g., Iceland) and is likely to become more commonplace in the next two decades.

To support the discovery of the genetic causes and contributors to common disease across all populations, researchers must be able to identify and access aggregated data from large-scale cohort population studies from diverse backgrounds, carried out by multiple distinct sites such as biobanks in the UK (UK BioBank, Generation Scotland), China (China Ka-doorie Biobank), the US (NIH All of Us Research Program), and Japan (Tohoku Medical Megabank, Japanese BioBank); and whole population cohorts in Iceland (deCODE), Estonia (Estonian Genome Project), and Finland (FinnGen). Doing so requires the data to be harmonized across all sites using common data models and terminologies. Furthermore,since genomic datasets of this scale are too large to download and manipulate at individual sites, researchers must be able to bring analytical tools to the data, regardless of their location.

Protocols are needed to deploy these tools consistently and effectively across distinct federated sites. GA4GH products support this critical type of biological study across the typical research life cycle from data discovery to analysis: (1) identify and access datasets relevant to a disease study (e.g., GA4GH Passports, DUO, multiple data discovery APIs), (2) access secure genotype and phenotype information on patients with related traits (e.g., Phenopackets, Data Repository Service [DRS] API, VRS, VA), and (3) remotely run analytical methods on data of interest (e.g., Task Execution Service [TES], Workflow Execution Service [WES] API, htsget API^12^), avoiding the need for inter-jurisdictional transfers and disparate regulatory requirements.

### Infectious disease

Genomics can be used to identify the infectious agents of disease with more confidence and precision than ever before, and at increasing speed, allowing treatments that can quickly resolve infections^78–80^ as well as identifying the evolution of new species that may evade antibiotics, antivirals, and vaccines. The main challenges to deployment of genomics in infectious disease care are managing cost and logistics, tracking disease progression and its characterization, achieving precise phenotypic prediction (e.g., antibiotic resistance), and harmonizing historical knowledge bases from non-genomic-based assays to integrate with contemporary genomic tests. The COVID-19 pandemic tested this infrastructure, with diagnostic testing becoming widespread, viral genomic sequencing enabling tracking of strains, and human genome sequencing of symptomatic individuals contributing to a better understanding of the basis of COVID-19 disease severity.^81^ Infectious disease genomic research and surveillance primarily rely on sequencing bacterial and viral pathogens and the organisms in which they are carried and transmitted. These genomes vary greatly in size, content, and associated metadata, so the standards and APIs created for human genomic data may be insufficient for infectious disease data. However, while the specific data standards needed to advance pathogen genomics differ from those in human genomics, there is still considerable overlap in the mechanics of sharing the data.

Through a variety of strategic alignments with organizations such as the Public Health Alliance for Genomic Epidemiology (PHA4GE; https://pha4ge.org/), the International COVID-19 Data Alliance (ICODA; http://www.icoda-research.org), and the European COVID19 data portal (http://www.covid19dataportal.org), GA4GH is working to ensure that the species-agnostic elements of genomic data sharing standards are transferred into the infectious disease community. In addition, some GA4GH standards have begun to explore how they should adapt to support infectious disease data; for example, the Phenopackets standard was improved to support case-level presentation for infectious diseases in 2020 in response to the COVID-19 pandemic. In addition, recently launched initiatives such as large-scale tuberculosis sequencing in several countries,^82^ rapid identification of Ebola and Zika virus strains,^83^ and tracing hospital outbreaks using genomics^84,85^ demonstrate a vibrant, functional interface between research, public health institutions, and clinical practice.

## Challenges to Secondary Use of Clinically Acquired Data

We envision the global clinical and research communities collaborating seamlessly in the context of practicing healthcare^86,87^ to enable a true “learning healthcare system” (LHS). The LHS concept has existed for over a decade;^88,89^ however, implementation is still in its infancy, facing several barriers.^90^ Some useful implementations are found across medicine,^91–94^ including genomic medicine.^95^ Increasing numbers of institutions and countries have begun biobanks, in many cases connected to their healthcare system (see Common/chronic disease above), providing fertile grounds on which to bring healthcare data— including clinical genomic data—into research.

To enable these efforts to reach their full potential, disparate systems must be able to share genomic and clinical data, requiring the community to overcome key challenges, particularly in the areas of infrastructure development, patient and physician incentives, ethics and regulation, privacy and security, and socio-cultural expectations (see Box 3). We believe these challenges can be overcome—but only if the genomics and healthcare communities commit to broad-based advocacy and coordinated efforts worldwide.

This has already been successfully modeled through the Clinical Genome Resource (ClinGen; a GA4GH Driver Project), where healthcare providers, clinical laboratory staff, and researchers work together to develop standards for gene and variant curation, share underlying evidence, and then apply that evidence through a consensus-driven process to classify genes and variants which are made freely accessible to the broader community to support both research and clinical care.^96,97^

### Developing clinical data standards

Much of the clinical data contained within healthcare are not encoded in a standardized format.^98^ Multiple electronic health record (EHR) vendors exist today and are highly proprietary in their technical structures, making standardization across EHRs and with downstream research systems difficult. Although data recorded in EHRs often use standardized clinical terminologies (e.g., ICD, SNOMED CT), the intent of these systems is generally to present clinical information on individuals to healthcare providers and, in some regions, facilitate billing practices. This presents a challenge for secondary users, where it is difficult to make accurate, population-scale conclusions, often requiring extensive efforts to understand practices and generate useful research data.^99^ In order to promote adoption of standardized formats in research and ultimately within EHRs, GA4GH is developing standardized information models (e.g., Phenopackets, Pedigree) to describe clinical phenotypes and family histories. Standardizing the representation of phenotype and pedigree information will allow patients, care providers, and researchers to share this information more easily between healthcare and research systems and enable software tools to use this information to improve genome analysis and diagnosis.

### Incentivizing and facilitating data sharing in healthcare

Resource limitations for healthcare providers and patients also impact their ability to share valuable clinical data. Some healthcare institutions (e.g., NHS England [https://www.england.nhs.uk/genomics/nhs-genomic-med-service], Dana-Farber Cancer Institute [http://www.dana-farber.org/for-patients-and-families/becoming-a-patient/preparing-for-your-first-appointment/checklist-for-new-adult-patients], Danish healthcare^100^) have built layered consent procedures into the regular routine of medical practice.^101^ Others support parallel biobanking efforts to separately consent patients for research.^102–106^ Still others have built this into their operations as an inherent part of the healthcare system.^100^ Further incentives can be built if providers can experience the direct benefits of research. For example, the clinical laboratory genetic testing industry largely participates voluntarily in data sharing through ClinVar, in part because they directly benefit from accurate variant interpretation.^50,107,108^ Several laboratories also joined when the US insurance industry began requiring submission as a condition of test reimbursement.^109^ However, despite progress in the sharing of variant knowledge, additional incentives and infrastructure are needed to support access to case-level results (e.g., variants interpreted for a patient indication) as well as full sequencing data, along with rich clinical phenotypes. Currently, most genetic test results are returned through PDF-based reports or accessed through external portals outside the medical system. Although standards exist for the exchange of genetic test results (see, for example, HL7’s guide in the web resources),^110^ robust standards that capture highly detailed, discrete genomic data are still under development. Adoption of those standards has been motivated by the implementation of downstream clinical decision support,^111–113^ but more incentives and infrastructure will be needed.

To date, GA4GH has worked on maintaining and evolving standardized file formats for raw and annotated genomic data (SAM, BAM, CRAM, VCF/BCF); individual variant representation and interpretation (VRS, VA); and transmission of individual phenotype data and interpreted results (Phenopackets), all of which are critical for the evolving use of genomics in healthcare systems—particularly clinical laboratory workflows to share genomic data and genetic testing results. Future areas of development include better representation of structural variants, unambiguous representation of complex multi-allelic loci, and research into new, more scalable formats for storing and exchanging genetic variation. Population-scale sequencing programs in which healthcare systems share clinical genomic data for research are unlikely to allow large-scale aggregation of data to migrate beyond national boundaries, but federated analysis—in which analytical algorithms or queries are brought to the data in its location without data egress—is feasible and is a major area of focus of GA4GH’s standards development.

### Ethics and regulation

Ethical considerations for patients and populations, together with responsible regulation, are essential for healthcare-funded genomics, which involves complex national regulation and legislation. Different countries and institutions have individual values and policies that relate to allowing access to personal information, with some embracing more open regulatory norms and systems on data collection, access, and sharing, and others being more restrictive. Nevertheless, most systems have some mechanism for researchers to access both research and clinical data. The GA4GH Regulatory and Ethics Work Stream (REWS) develops ready-to-use policy guidance to support responsible, international genomic and health-related data sharing. In Box 4, we list central components of the GA4GH Regulatory & Ethics Toolkit, including policies, consent tools, and data access guidance. The REWS also reviews all GA4GH technical standards for consideration of any regulatory or ethics issues that may be relevant.

The first REWS product was the GA4GH Framework for Responsible Sharing of Genomic and Health-Related Data, ^115^ which is built on the human right to benefit from scientific progress and its applications, as well as privacy, non-discrimination, and procedural fairness. It provides guidance for the responsible sharing of human genomic and health-related data, including personal health data and other types of data that may have predictive power in relation tohealth. The Framework has now been translated into 14 languages and has been used to inform local data sharing approaches around the globe, including, for example, the World Economic Forum,^116^ the Academy of Science of South Africa,^117^ DNA.Land, Health Data Research UK,^118^ and the Horizon-2020 CORBEL project.^119^ Keeping the fundamental human right to benefit from science at the heart of clinical and genomic data sharing ensures a universal approach to balancing the benefits and potential risks. We believe that most healthcare system actors can ultimately participate in responsible, worldwide data sharing while remaining compliant with applicable laws and institutional policies.

### Privacy and security

Federating large volumes of sensitive clinical and genomic data across internationally distributed virtual computing environments presents formidable challenges in assuring data integrity, service availability, and individual privacy. Some of these challenges call for innovative application of well-established security standards, frameworks, and protocols—such as identity federation on a global scale—and some GA4GH standards already do so (e.g., crypt4GH, Authentication & Authorization Infrastructure [AAI] / Passports). Another crucial challenge is to enable secure, privacy-preserving federated analysis, wherein researchers can extract information without having to transfer raw data. This evolution is key to foster inter-institutional and international collaboration and will be a strong incentive to improve ontology homogeneity. Several technical solutions are available, either based on hardware devices or on software algorithms. The former are computationally efficient, but require trusting a vendor and are prone to side-channel attacks. The latter are computationally slower, but are mathematically proven and are a better response to GA4GH expectations. Recent results have demonstrated the effectiveness of a software-based approach (a combination of homomorphic cryptography and secure multiparty computation called “Multi-party Homomorphic Encryption” or MHE); these approaches have been positioned with respect to the GDPR.^120,121^ One of the major strengths of MHE is that partial aggregates can be considered to be anonymized and not just pseudonymous, in the sense of GDPR, and thus potentially obviating the need for data transfer and use agreements (DTUAs).

### Societal challenges

Societal challenges of allowing access to genomic data within the healthcare ecosystem include maintaining public trust, overcoming differences in objectives and methods between research and healthcare, and breaking down unproductive divides between disciplines. Our vision for healthcare data ecosystems is one in which vetted researchers around the world can, with appropriate oversight and policy enforcement, gain access to human health data for the benefit of patients. GA4GH has defined the core elements of responsible data sharing, including transparency, accountability, recognition, and attribution as well as sanctions for misuse which form a framework to respect and maintain the trust of participants.^122^ In particular, the GA4GH Engagement Framework (see Box 4) further assists researchers in designing and understanding engagement with public, patient, and participant stakeholders through the central themes of fairness, context, heterogeneity, and the recognition of tensions. Through the implementation arm of GA4GH, the Genomics in Health Implementation Forum (https://www.ga4gh.org/implementation) described below and other engagement efforts, GA4GH is tackling the broader societal implementation issues including education and engagement of the public, healthcare providers, and regulators in order to build trust within the community. The GA4GH “Your DNA, Your Say” survey, an effort to gather international public attitudes toward genomic data sharing, has provided an evidence base for understanding which factors are important to maintaining public trust in the generation and sharing of genomic data, as well as how concerns differ according to geography.^123,124^ These findings help ensure that GA4GH’s work can enhance the public trust in a global context upon which the future of genomics depends.

## Connecting Standards for Implementation

With more than 30 GA4GH standards approved, and dozens of production-ready implementations of those standards deployed around the world, GA4GH is now shifting its focus toward demonstrating how standards can work together to provide seamless support of genomic activities. Interconnected standards that are compatible and interoperable with each other and are hardened for real-world use will enable solutions for federated analyses across platforms and use cases. To drive this effort, GA4GH has established the Federated Analysis System Project (FASP), which aims to demonstrate how GA4GH APIs, when used in concert, can support real-world, scientific use cases (see https://www.ga4gh.org/genomic-data-toolkit/2020-connection-demos/). A key outcome of FASP is a series of scripts that represent working examples of clients accessing real-world GA4GH-compatible services to solve a spectrum of challenges across the search-access-analyze workflow. The scripts illustrate how these services have adopted GA4GH standards to solve challenges, such as dataset discoverability and controlled data access, in order to drive larger scale and more powerful analyses.

By developing working implementations of GA4GH standards that are pressure tested in real world scenarios, the FASP team has identified specific areas of improvement within the standards. As a result of this work, new features will be added to existing GA4GH specifications to further facilitate secure, real-world federated data sharing and analysis. Most notably, the group is working toward a standardized solution for using a GA4GH Passport to access a controlled access dataset from a Data Repository Service (DRS), while fulfilling robust security requirements, such as preventing escalation of privilege. These efforts will be critical to support access to valuable datasets across the globe.

## GA4GH Starter Kit

To date, GA4GH has primarily focused on overcoming the challenges of enabling interoperability within new initiatives built on a foundation of cloud infrastructure. However, an additional—and potentially more significant—challenge is bringing high-performance computing (HPC) infrastructures that are not already focused on cloud interoperability into the federated network envisioned by this community.

While more ambitious goals are on the horizon for connecting and extending GA4GH standards (e.g., discovery of datasets; matching requests, analyses, and datasets; describing phenotypes; reporting on variants), FASP has shown through its real-world demonstrations of access across distributed but interoperable datasets that the initial groundwork for federated analysis is now in place. The Data Repository Service (DRS) allows data custodians to make controlled access data available at multiple sites; the Workflow Execution and Task Execution Services (WES & TES) allow researchers to encapsulate and run analyses on those data; and AAI and Passports allow for federated authorization and authentication, streamlining the data access process for both researchers and data custodians.

In 2021, GA4GH has begun to develop the GA4GH Starter Kit, a set of open source reference implementations (for example, code bases that demonstrate the standards working in practice), to help ensure existing HPC environments can interoperate with the wider GA4GH network. These resources consist of “plug- and-play” code that any institution (cloud-based or HPC) can use to quickly achieve GA4GH compatibility and will facilitate the progressive movement of established large-scale systems toward interoperability. In addition, a testing suite will be developed to ensure deployments of both reference and non-reference implementations are compliant to their respective GA4GH specifications.

## Genomics in Health Implementation Forum

Once standards have been piloted in real-world Driver Project settings and shown to enable true federated analysis in FASP, they can begin to be promoted more broadly in the research and clinical genomics communities. Launched in 2020, the Genomics in Health Implementation Forum (GHIF) brings together a group of national-scale genomic data initiatives to share resources, experiences, and best practices for implementing GA4GH standards, as well as broader experience in rolling out national and international data sharing activities. GHIF aims to support more accurate data interpretation and disease diagnosis plus other innovative solutions across healthcare through global cooperation in data sharing and clinical implementation of genomics.

Broad uptake of GA4GH standards among GHIF members— which include both GA4GH Driver Projects as well as other national and multi-national initiatives (see https://ga4gh.org/implementation for full list)—will provide strong evidence that GA4GH standards are supporting the community’s actual data sharing needs.

Implementation of GA4GH policies and standards throughout the scientific and healthcare communities will allow researchers to access data across the globe—a critical step toward answering otherwise impenetrable questions about disease and basic human biology. As the volume of genomic and health-related data grows exponentially around the world, researchers, clinicians, and bioinformaticians have a responsibility to make that data appropriately accessible and to use it to realize benefits for all humans everywhere. The promise of genomic medicine lies at a crossroads that depends on harmonization across the global community to significantly enhance human health and medicine. We believe that GA4GH, by embracing collaborative innovation and knowledge exchange, is well poised to meet this challenge.

## Supplementary Material

S1

S2

## Figures and Tables

**Table 1:** GA4GH toolkit

Relevant standards	URL	Type	Target user	Purpose
**Identify and access datasets relevant to a disease study**				
Beacon API^8^	https://app.swaggerhub.com/apis/ELIXIR-Finland/ga-4_gh_beacon_api_specification/1.0.0-rc1	API	data custodians, researchers (via research infrastructures), identity provider services	The Beacon protocol defines an open standard for genomics data discovery. It provides a framework for public web services responding to queries against genomic data collections, for instance from population-based or disease-specific genome repositories. Beacon is designed to (1) focus on robustness and easy implementation, (2) be maintained by individual organizations and assembled into a federated network, (3) be general-purpose and able to be used to report on any variant collection, (4) provide a boolean (or quantitative) answer about the observation of a variant, and (5) protect privacy, with queries not returning information about single individuals. A new version of the API will include support for more granular control based on a user’s identity authorization and will enable discovery of cohorts, cases (patients), biological samples, and genomic variants and associated knowledge. More details can be found on the Beacon Project website.
Data Connect	https://github.com/ga4gh-discovery/data-connect	API	data custodians, researchers, and API & tool developers	Data Connect is a specification for discovery and search of biomedical data, which provides a mechanism for describing data and its data model, and for searching data within the given data model. The primary container for data in Data Connect is the table. Tables contain rows of data, where each row is a JSON object with key/value pairs. The table describes the structure of its row objects using JSON Schema (https://json-schema.org/). Row attributes can take on any legal JSON value, e.g., numbers, strings, booleans, nulls, arrays, and nested JSON objects. The API supports browsing and discovery of data models and table metadata, listing table data, and optionally searching table data using arbitrarily complex expressions including joins and aggregations. The query language is SQL with domain-specific functions to facilitate informative typing of the result fields. Data publishers can wrap existing data storage and retrieval systems in the Data Connect API or may choose to publish data directly as static files in the Data Connect JSON format. Data consumers can use Data Connect via graphical data discovery and exploration built upon the API, via command line tools (interactively or in batch workflows), and directly as an API in custom analysis programs. More information can be found in the specification (https://github.com/ga4gh-discovery/data-connect/blob/master/SPEC.md).
Data Use Ontology^9^	http://purl.obolibrary.org/obo/duo.owl	Data Model / Ontology	data custodians, researchers, DACs	The Data Use Ontology (DUO) is a hierarchical vocabulary of terms describing data use permissions and modifiers, in particular for research data in the health/clinical/biomedical domain. The GA4GH DUO standard allows large genomics and health data repositories to consistently annotate their datasets, ensuring a shared, machine readable, representation of data access conditions, and making them automatically discoverable based on a researcher’s authorization level or intended use. Reference implementations are available at Broad’s FireCloud - Data Library Broad’s DUOS (Data Use Oversight System) - Data Catalog European Genome-Phenome Archive. DUO is based on the OBO Foundry principles (http://www.obofoundry.org/principles/fp-000-summary.html) and developed using the W3C Web Ontology Language. DUO can be browsed online via the Ontology Lookup Service or Ontobee. It has been registered with the OBO Foundry (http://obofoundry.org/ontology/duo.html).
GA4GH Passports^10^	https://github.com/ga4gh-duri/ga4gh-duri.github.io/blob/master/researcher_ids/ga4gh_passport_v1.md	API / Data Model	data custodians, researchers, DACs, clinicians, API and tool developers	The GA4GH Passport specification aims to support data access policies within current and evolving data access governance systems. This specification defines Passports and Passport Visas as the standard way of communicating a user’s data access authorizations based on either their role (e.g., researcher), affiliation, or access status. Passport Visas from trusted organizations can therefore express data access authorizations that require either a registration process (for the Registered Access data access model^11^) or custom data access approval (such as the Controlled Access applications used for many datasets).
Service Info	https://github.com/ga4gh-discovery/ga4gh-service-info	API	API and tool developers	Service discovery is at the root of any computational workflow using web-based APIs. Traditionally, this is hard-coded into workflows, and discovery is a manual process. Service Info provides a way for an API to expose a set of metadata to help discovery and aggregation of services via computational methods. It also allows a server/implementation to describe its capabilities and limitations. Service-info is described in GA4GH OpenAPI specification, which can be visualized using Swagger Editor (https://editor.swagger.io/?url=https://raw.githubusercontent.com/ga4gh-discovery/ga4gh-service-info/develop/service-info.yaml).
Service Registry	https://github.com/ga4gh-discovery/ga4gh-service-registry	API	API and tool developers	Service registry is a GA4GH service providing information about other GA4GH services, primarily for the purpose of organizing services into networks or groups and service discovery across organizational boundaries. Information about the individual services in the registry is described in the complementary Service Info specification (see above). The Service Registry specification is useful when dealing with technologies that handle multiple GA4GH services. Common use cases include creating networks or groups of services of a certain type (e.g., Beacon Network searches networks of Beacon services across multiple organizations, a workflow can be executed by a specific group of Workflow Execution Services, or Data Connect search on biomedical data is federated across a set of nodes), or a certain host (e.g., an organization provides implementations of Beacon, Data Connect, and Data Repository Service APIs, or a server hosts an implementation of refget and htsget APIs).
Remotely run analytical methods on data of interest				
htsget^12^	samtools.github.io/hts-specs/htsget.html	API	API and tool developers, researchers	htsget is a data retrieval API that bridges from existing genomics file formats to a client/server model with the following features: Incumbent data formats (BAM, CRAM, VCF) are preferred initially, with a future path to others. Multiple server implementations are supported, including those that do format transcoding on the fly, and those that return essentially unaltered filesystem data. Multiple use cases are supported, including access to small subsets of genomic data (e.g., for browsing a given region) and to full genomes (e.g., for calling variants).
refget^13^	samtools.github.io/hts-specs/refget.html	API	API and tool developers, researchers	Refget (https://w3id.org/ga4gh/refget) is an API and mechanism for generating identifiers for reference sequences and retrieving sequences via API. The refget identifier is derived from sequence content directly and therefore does not rely on a central issuing authority. This allows downstream clients to unambiguously refer to a reference sequence and to retrieve said sequence. The refget API can also provide subsequences and metadata pertaining to the checksum identifier. A refget server can host any number of reference sequences of any type, e.g., genomic DNA or protein sequences. The refget protocol is a fundamental building block of the CRAM specification. An OpenAPI description of this specification is available and describes the 1.0.0 version (https://github.com/samtools/hts-specs/blob/master/pub/refget-openapi.yaml). Implementors can check if their refget implementations conform to the specification by using our compliance suite (https://github.com/ga4gh/refget-compliance-suite). A summary of all known public implementations is available from our compliance report website.
Task Execution Service (TES)	https://github.com/ga4gh/task-execution-schemas	API	API and tool developers, researchers, academic institutions	The Task Execution Service (TES) API is a standardized schema and API for describing and executing batch execution tasks. A task defines a set of input files, a set of containers and commands to run, a set of output files, and some additional logging and metadata. TES servers accept task documents and execute them asynchronously on available compute resources. A TES server could be built on top of a traditional HPC queuing system, such as Grid Engine, Slurm, or cloud style compute systems such as AWS Batch or Kubernetes.
Tool Registry Service (TRS)	https://github.com/ga4gh/tool-registry-service-schemas	API	API and tool developers, researchers, academic institutions	The GA4GH Tool Registry (TRS) API aims to provide a standardized way to describe the availability of tools and workflows. In this way, multiple repositories that share Docker-based tools and workflows (based on Common Workflow Language [CWL], Workflow Description Language [WDL], Nextflow, or Galaxy) can consistently interact, search, and retrieve information from one another. The end goal is to make it much easier to share scientific tools and workflows, enhancing our ability to make research reproducible, shareable, and transparent. To access the specification, users can: view the human-readable Reference Documentation explore the specification in the Swagger Editor preview documentation from the gh-openapi-docs for the development branch at https://ga4gh.github.io/tool-registry-service-schemas/preview/develop/docs/index.html
Workflow Execution Service (WES)	https://github.com/ga4gh/workflow-execution-service-schemas	API	API and tool developers, researchers, academic institutions	The Workflow Execution Service (WES) API describes a standard programmatic way to run and manage workflows. Having this standard API supported by multiple execution engines will let people run the same workflow using various execution platforms running on various clouds/environments. Key features include: (1) ability to request a workflow run using CWL or WDL; (2) ability to parameterize that workflow using a JSON schema; and (3) ability to get information about running workflows.
Securely access genotype and phenotype information on patients with related traits				
Authentication & Authorisation Infrastructure (AAI)	https://github.com/ga4gh/data-security/blob/master/AAI/AAIConnectProfile.md	Guide	API and tool developers	The GA4GH Authentication & Authorisation Infrastructure (AAI) specification profiles the OpenID Connect (OIDC) protocol to provide a federated (multilateral) authentication and authorization infrastructure for greater interoperability between genomics institutions in a manner specifically applicable to (but not limited to) the sharing of restricted datasets. In particular, this specification introduces a JSON Web Token (JWT) syntax for an access token to enable an OIDC provider (called a Broker) to allow a downstream access token consumer (called a Claim Clearinghouse) to locate the Broker’s /userinfo endpoint as a means to fetch GA4GH Claims. This specification is suggested to be used together with others that specify the syntax and semantics of the GA4GH Claims exchanged.
Cloud Security and Privacy Policy v1.0	https://docs.google.com/document/d/1cBTwtetnsvO2vU3HVwLTLaC9H_ya-4MjZUa_g_xzOBg/edit	Guide	anyone handling sensitive data in a cloud infrastructure.	An increasing number of GA4GH projects rely on Cloud services to pursue their goals, and the GA4GH Cloud Work Stream is working on several products to make the GA4GH community take full advantage of the Cloud paradigm. However, the use of the Cloud poses significant security and privacy challenges that need to be carefully evaluated and addressed. The purpose of the Cloud Security and Privacy Policy is to outline a common security technology framework that can be used to systematically assess the products developed by the CWS from a security perspective. Product developers and reviewers can leverage the information contained herein to identify requirements, threats, and countermeasures related to the products they are working on, thus facilitating the production of secure standards.
CRAM^14^	samtools.github.io/hts-specs/CRAMv3.pdf	File Format	API and tool developers, researchers	The CRAM file format holds DNA sequencing records. It has the following major objectives: Significantly better lossless compression than BAM To permit simple and lossless transformations to and from BAM files Support for controlled loss of data The first two objectives allow users to take immediate advantage of the CRAM format while offering a smooth transition path from using BAM files. The third objective supports the exploration of different lossy compression strategies and provides a framework in which to effect these choices. Data in CRAM is stored in a columnar fashion, with each column being compressed with either a general-purpose compressor or a custom method. If aligned, sequences may be stored as differences against a reference sequence, which is optionally stored within the CRAM file. External references may be either a local file or obtained remotely via the refget API. Data may be retrieved either as whole alignment records, or selectively only for the fields (columns) required.
Crypt4GH^15^	samtools.github.io/hts-specs/crypt4gh.pdf	File Format	API and tool developers, data generators, researchers, clinicians, data custodians	By its nature, genomic data can include information of a confidential nature about the health of individuals. It is important that such information is not accidentally disclosed. One part of the defense against such disclosure is to, as much as possible, keep the data in an encrypted format. The Crypt4GH specification describes a file format that can be used to store data in an encrypted state. Existing applications can, with minimal modification, read and write data in the encrypted format. The choice of encryption also allows the encrypted data to be read starting from any location, facilitating indexed access to files. The format has the following properties: Confidentiality: Data stored in the file are readable only by holders of the correct secret decryption key. The format does not hide the length of the encrypted file, although it is possible to pad some file structures to obscure the length. Integrity: Data are stored in a series of 64 kilobyte blocks, each of which includes a message authentication code (MAC). At tempts to change the data in a block will make the MAC invalid; it is not possible to recalculate the MAC without knowing the key used to encrypt the file. The format only protects the contents of each individual block. It does not protect against insertion, removal, or reordering of entire blocks. Authentication: The format does not provide any way of authenticating files. Crypt4GH may be used with any data file or stream, but one usage is encryption of BAM, CRAM, VCF, and BCF data within the htsget API while still retaining full random access.
Data Repository Service (DRS)	https://github.com/ga4gh/data-repository-service-schemas	API	API and tool developers, researchers, academic institutions	The Data Repository Service (DRS) API provides a generic interface to data repositories so data consumers, including workflow systems, can access data objects in a single, standard way regardless of where they are stored and how they are managed. The primary functionality of DRS is to map a logical ID to a means for physically retrieving the data represented by the ID. The DRS specification describes the characteristics of those IDs, the types of data supported, how they can be pointed to using URIs, and how clients can use these URIs to ultimately make successful DRS API requests. The specification also describes the DRS API in detail and provides information on the specific endpoints, request formats, and responses. This specification is intended for developers of DRS-compatible services and of clients that will call these DRS services.
Data Security Infrastructure Policy (DSIP)	https://github.com/ga4gh/data-security/blob/master/DSIP/DSIP_v4.0.md	Policy Framework	data protection authorities	The Data Security Infrastructure Policy (DSIP) describes the data security infrastructure recommended for stakeholders in the GA4GH community. It is not meant to be a normative document, but rather a set of recommendations and best practices to enable a secure data sharing and processing ecosystem. However, it does not claim to be exhaustive, and additional precautions other than the ones collected in the policy might have to be taken to be compliant with national/regional legislations. As a living document, the DSIP will be revised and updated over time, in response to changes in the GA4GH Privacy and Security Policy, and as technology and biomedical science continue to advance.
Machine Readable Consent Guidance (MRCG) v1.0	https://www.ga4gh.org/wp-content/uploads/Machine-readable-Consent-Guidance_6JUL2020-1.pdf	Guide	researchers, institutional review boards/research ethics committees (international and national), research ethics policy makers, data generators, funding agencies	The Machine Readable Consent Guidance (MRCG) provides standardized consent clauses and supporting information to enable the development of consent forms that map unambiguously to the GA4GH Data Use Ontology (DUO). Integrating DUO into consent forms thereby facilitates data discovery and data access requests and approvals, maximizing data sharing, integration, and re-use while respecting the autonomy of data subjects. MRCG implementations include the Broad Data Use Oversight System (DUOS)^16^ and the Australian Genomics dynamic consent participant platform, CTRL.
Pedigree V1	https://github.com/GA4GH-Pedigree-Standard/pedigree	Data Model / Ontology	clinicians, researchers, API and tool developers, data generators, EHR vendors	Family health history is an important aspect in both genomic research and patient care. The GA4GH pedigree standard is an object-oriented graph-based model to represent family health history and pedigree information. It is intended to fit within the structure of other standards like HL7 FHIR and Phenopackets and enable the computable exchange of family health history as well as representation of larger, more complex families. Computable representation of family structure will allow patients, physicians, and researchers to share this information more easily between healthcare systems and help software tools use this information to improve genomic analysis and diagnosis. The draft model can be found on Github along with a Family History Relations Ontology and draft FHIR implementation guide. A draft recommendation for a minimal dataset of family health history (https://docs.google.com/document/d/1UAtSLBEQ_7ePRLvDPRpoFpiXnl6VQEJXL2eQByEmfGY/edit?usp=sharing) was developed as a foundation of these efforts.
Phenopackets	http://phenopackets.org	Data Model / Ontology	data generators, data custodians, researchers, clinicians, API and tool developers	The Phenopacket specification is an open machine-readable schema that supports the global exchange of disease and phenotype information to improve our ability to diagnose and conduct research on all types of diseases, including cancer and rare disease. A Phenopacket links detailed phenotypic descriptions with disease, patient, and genetic information, enabling clinicians, biologists, and disease and drug researchers to build more complete models of disease. Version 2 of the standard, released in June 2021, expands on the previous version to include better representation of the time course of disease, treatment, and COVID-19 and cancer-related data. The schema, as well as source code in Java, C++, and Python, are available from the phenopacket-schema GitHub repository.
RNAget	https://ga4gh-rnaseq.github.io/schema/docs/index.html	API	Data generators, data custodians, researchers, tool developers	The RNAget API describes a common set of endpoints for search and retrieval of processed RNA data. This currently includes feature level expression data from RNA-seq type assays and signal data over a range of bases from ChIP-seq, methylation, or similar epigenetic experiments. By using these common endpoints, data providers make it easier for client software to access their data with minimal or no modifications to underlying code. This improves interoperability with other compliant data providers and makes it easier for investigators to retrieve and compare data from multiple sites. For the software developer, these common endpoints and patterns make it easier to access multiple compliant server sites with the same client software. This reduces development time which may have otherwise been spent writing parsers and custom request generators. Using the API, it becomes much easier to write software to conduct comparisons, data mingling, or other analyses on data retrieved from multiple, potentially geographically dispersed data servers. The OpenAPI description of the specification can be used with code generators like OpenAPI Generator. The testing and compliance page includes a list of example server implementations which can be used as is or as a starting point. A custom solution can be implemented to link the API endpoints and queries to a local data backend (of any desired type) serving the data.
SAM and BAM^17^	samtools.github.io/hts-specs/SAMv1.pdf	File Format	researchers	SAM, or Sequence Alignment/Map format, is a format for storing primary DNA sequencing records. These are typically aligned and sorted by genomic coordinate, but unaligned data can also be represented. SAM is a TAB-delimited text format consisting of a header meta-data section and an alignment section. The BAM format is a binary serialization of SAM for more efficient access. SAM and BAM support full random access, selected by genomic region. The SAMtags document defines the optional per-record annotations. These are also used by the CRAM specification.
Variant Annotation	https://github.com/ga4gh/va-spec	Data Model / Modeling Framework	API and tool developers	Variant annotations are structured data object that holds a central piece of knowledge about a genetic variation, along with metadata supporting its interpretation and use. A given variant annotation may describe knowledge about its molecular consequence, functional impact on gene function, population frequency, pathogenicity for a given disease, or impact on therapeutic response to a particular treatment. The GA4GH VA-Specification will define an extensible data model for representation and exchange these and other diverse kinds of variant annotations. It will provide machine-readable messaging specifications to support sharing and validation of data through APIs and other exchange mechanisms. It will also provide a formal framework for defining custom extensions to the core model - allowing community-driven development of VA-based data models for new data types and use cases. A more detailed description of these components can be found online. The VA-Spec is being authored by a partnership among national resource providers and major public initiatives within GA4GH. It has been informed by and will be tested in diverse, established, and actively developed Driver Projects, including ClinGen, VICC, Genomics England, the Monarch Initiative, BRCA Exchange, and Australian Genomics. In these contexts, it will be used to support different types of tools and information systems, including variant curation tools and interpretation platforms (e.g., ClinGen, CIViC, Genomics England), variant annotation services (e.g., CellBase), knowledge aggregators/portals (e.g., BRCA Exchange, Monarch Initiative), matchmaking applications (e.g., Matchmaker Exchange), and clinical information systems and decision support tools.
Variation Representation^18^	https://vrs.ga4gh.org	Data Model & terminology	data generators, API and tool developers, data custodians	Maximizing the personal, public, research, and clinical value of genomic information will require that clinicians, researchers, and testing laboratories exchange genetic variation data reliably. The Variation Representation Specification (VRS, pronounced “verse”) — written by a partnership among national information resource providers, major public initiatives, and diagnostic testing laboratories — is an open specification to standardize the exchange of variation data. The primary contributions of VRS include (1) terminology and an information model, (2) a machine readable schema, (3) conventions that promote reliable data sharing, (4) globally unique computed identifiers, and (5) a Python implementation (available at vrs-python) that demonstrates the above schema and algorithms and supports translation of existing variant representation schemes into VRS for use in genomic data sharing. It may be used as the basis for development in Python, but it is not required in order to use VRS. The machine-readable schema definitions and example code are available online at the VRS repository. Readers may wish to view a complete example before reading the specification. For a discussion of VRS with respect to existing standards, such as HGVS, SPDI, and VCF, see “Relationship of VRS to existing standards,” an appendix to the specification documentation.
VCF/BCF^19^	samtools.github.io/hts-specs/VCFv4.3.pdf	File Format	researchers	The variant call format (VCF) is a generic format for storing DNA polymorphism data such as single nucleotide polymorphisms (SNPs), insertions, deletions, and structural variants, together with rich annotations. VCF may hold data for multiple samples within the same file. The specification contains the header meta-data fields, a series of mandatory columns describing the variants, and details of the optional annotations which are either per-site or per-sample. VCF and its binary counterpart, BCF, is usually stored in a compressed manner and can be indexed for fast data retrieval of variants from a range of positions on the reference genome.

The GA4GH Toolkit outlines a suite of secure standards and frameworks that will enable more meaningful research and patient data harmonization and sharing. This suite addresses a variety of challenges across the data sharing life cycle and is applicable across the world’s accessible medical and patient-centered systems, knowledgebases, and raw data sources. All standards are subject to the GA4GH Copyright Policy (https://www.ga4gh.org/wp-content/uploads/GA4GH-Copyright-Policy-Updated-Formatting.pdf) and should be made available under an open source license such as the Apache 2.0 license for software.

**Table 2:** GA4GH Driver Projects

Driver Project	URL	Location	Thematic area*	Current size	Data type(s) collected	Data hosting model(s)	Data access model(s)	Implementations / deployments of GA4GH standards
All of Us Research Program	https://allofus.nih.gov/	US	RD, Ca, CT	100k whole-genome sequences (planning for 1 million)	WGS, WES	centralized	cloud	CRAM, DRS (forthcoming), htsget (forthcoming), Passports (forthcoming), TRS (forthcoming), and WES (forthcoming)
Australian Genomics	https://www.australiangenomics.org.au/	Australia	RD, Ca, CT	13,500 whole-genome sequences across all pilots	WGS, WES, panels, phenotype	centralized	cloud	Beacon V1, CRAM, Crypt4GH, DRS (forthcoming), DUO, htsget, MRCG (forthcoming), Passports (forthcoming), refget
Autism Sharing Initiative	https://www.autismsharinginitiative.org/	international	CT	11,316 whole-genome sequences (estimating 15k by 2025)	WGS	distributed	federated analysis	AAI (forthcoming), Beacon V1 (forthcoming), CRAM (forthcoming), Data Connect, DRS (forthcoming), DUO (forthcoming), Passports (forthcoming), Service Registry / Info, TRS (forthcoming), WES (forthcoming)
BRCA Exchange	http://www.brcaexchange.org	international	RD, Ca	66,657 variants	genetic variant pathogenicity assertions and supporting evidence	centralized	public	Beacon V1, VA (forthcoming), VRS, WES (forthcoming)
CanDIG	https://www.distributedgenomics.ca/	Canada	RD, Ca, CT, Bio	1,700 data records	WGS tumor/normal and whole transcriptome for cancer; WGS for COVID; clinical phenotype	distributed	federated analysis	Beacon V1, CRAM, DRS, DUO, htsget, Phenopackets, refget (forthcoming), RNAGet, Service Registry / Info (forthcoming), VRS (forthcoming), WES (forthcoming)
ClinGen	https://www.clinicalgenome.org/	US	RD	2,077 unique genes with at least one curation and 2,417 unique variants with at least one curation	genetic and experimental evidence	centralized	public	VA (forthcoming), VRS
ELIXIR	https://elixir-europe.org/	Europe	RD, Ca, CT, Bio	23 national nodes hold a variety of data types and run multiple services, some listed within this table (e.g., EGA). For a list of ELIXIR Core Data Resources, see https://elixir-europe.org/platforms/data/core-data-resources		distributed	download (also exploring Cloud)	AAI, Beacon V1, Crypt4GH, DRS, DUO, htsget, Passports, Phenopackets, refget, RNAGet, Service Registry / Info, TES, TRS, WES
ENA / EVA / EGA	https://www.ebi.ac.uk/ena,	Europe	RD, Ca, CT, Bio	EGA - 700k data records	EGA - WGS, WES, RNaseq, epigenetics, genotyping, transcriptome, singlecell seq, healthy and disease cohorts	distributed	download (also exploring Distributed Cloud)	Crypt4GH, htsget AAI, Passports, DUO
EpiShare	https://epishare-project.org/	international	Bio	~2,800 data records	FASTQ, CRAM/BAM, bigwig, bigbed for epigenomics experiments	distributed	federated analysis	CRAM (forthcoming), DRS, DUO, htsget (forthcoming), Phenopackets, RNAGet, Service Registry / Info, WES
EUCANCan	http://www.eucancan.com	international	Ca	data from 35 different sources including human, model, and non-model organisms	whole-genome, whole-exome, and whole-transcriptome sequence data	distributed	Cloud and federated analysis	AAI (forthcoming), Beacon V1 (forthcoming), CRAM (forthcoming), Data Connect (forthcoming), DRS (forthcoming), Passports (forthcoming), Phenopackets (forthcoming), Service Registry / Info (forthcoming), TES (forthcoming), TRS (forthcoming), VRS (forthcoming), WES (forthcoming)
European Joint Programme on Rare Disease (EJP RD)	https://www.ejprarediseases.org/	Europe	RD	>130,000 data records across several resources hosting genomic human data, mainly the EGA, DECIPHER and the RD-Connect Genome-Phenome Analysis Platform	a mix of WGS, WES, plausibly pathogenic variants and phenotypic information	distributed across centralized resources	download and Cloud analysis	AAI (forthcoming), Beacon V1, CRAM, Crypt4GH, DRS (forthcoming), DUO, htsget, Passports, Phenopackets, Service Registry / Info, TES, TRS, WES
GEnome Medical Alliance Japan (GEM Japan)	https://www.amed.go.jp/en/aboutus/collaboration/ga4gh_gem_japan.html	Japan	RD, Ca, CT	24k WGS (aiming for 100k)	whole-genome sequencing, whole-exome sequencing, gene expression, panels, phenotypic	centralized	download (also exploring Cloud)	Beacon V1 (forthcoming), CRAM, DUO, Phenopackets (forthcoming)
Genomics England	https://www.genomicsengland.co.uk	UK	RD, Ca, CT	136K WGS, (estimating 450K WGS by 2024)	WGS	centralized	Cloud	AAI (forthcoming), CRAM, DRS (forthcoming), DUO (forthcoming), htsget, Passports (forthcoming), WES (forthcoming)
Human Cell Atlas	https://www.humancellatlas.org	International	RD, Ca, CT, Bio	1,300 donors	single-cell sequencing	centralized	public and Cloud	AAI, DRS, DUO (forthcoming), Passports (forthcoming), TES, TRS, WES
Human Heredity and Health in Africa (H3Africa)	https://h3africa.org/	Africa	CT, Bio	75,000 participants (across all projects)	whole-genome sequencing, whole-exome sequencing, gene expression, microbiome, imaging, phenotypic, environmental/lifestyle	centralized	download	AAI (forthcoming), Beacon V1, CRAM, Crypt4GH, Data Connect (forthcoming), DUO, Passports (forthcoming), Phenopackets (forthcoming), VRS (forthcoming)
International Cancer Genome Consortium (ICGC) Accelerating Research in Genomic Oncology (ARGO)	https://www.icgc-argo.org	international	Ca	100k Genomes	WGS, WES, RNA-Seq, phenotype	distributed	Cloud and federated analysis	AAI (forthcoming), Beacon V1, CRAM, Passports (forthcoming), TRS, WES
Matchmaker Exchange	https://www.matchmakerexchange.org	international	RD	>109K cases	WGS, WES	distributed	federated analysis	AAI (forthcoming), Beacon V1, CRAM, htsget, Phenopackets
Monarch Initiative	https://monarchinitiative.org/	international	RD, Ca, CT, Bio	N/A	gene, genotype, variant, disease, and phenotype data across many species in the tree of life, from over 30 data sources	centralized	public cloud	DUO (forthcoming), Passports (forthcoming), Phenopackets, VRS
National Cancer Institute Cancer Research Data Commons (NCI CRDC)	https://datascience.cancer.gov/data-commons	US	Ca	~100,000 data records (includes GDC)	whole-genome sequencing, whole-exome sequencing, gene expression, panels, phenotypic, biospecimen, imaging, proteomics	centralized	Cloud and federated analysis	CRAM, DRS, DUO (forthcoming), Passports (forthcoming), Service Registry / Info, WES
National Cancer Institute Genomic Data Commons (NCI GDC)	https://gdc.cancer.gov	US	Ca	83,700 cases	WGS, WXS, panel, RNA-seq, miRNA-seq, methylation array, genotyping array, diagnosis slides, tissue slides, ATAC-seq, scRNA-seq. Also clinical (phenotypic) and biospecimen information	centralized	download and Cloud	AAI (forthcoming), CRAM (forthcoming), DRS (forthcoming), DUO (forthcoming), Passports (forthcoming), Phenopackets (forthcoming), TES (forthcoming), TRS (forthcoming), VRS (forthcoming), WES (forthcoming)
Swiss Personalized Health Network (SPHN)	http://sphn.ch	Switzerland	RD, Ca, CT, Bio	24 health data projects across Switzerland	clinical phenotypic, clinical routine, omics (genomic, transcriptomic, proteomic, etc), cohort, and imaging data and expert variant curation	distributed	federated analysis	Beacon V1, DRS (forthcoming), htsget (forthcoming), Phenopackets, TES (forthcoming), WES (forthcoming)
Trans-Omics for Precision Medicine (TOPMed)	https://topmed.nhlbi.nih.gov	US	RD, Ca, CT, Bio	180k whole genome sequences (233k by 2025), 96k panels	WGS, RNA-seq, metabolome, methylome (MethylationEPIC ‘850K’), proteome (SomaScan and Olink), longitudinal epidemiology studies, disease-studies, environmental/lifestyle, imaging	centralized	cloud	AAI (forthcoming), CRAM, DRS, DUO, Passports (forthcoming), Service Registry / Info (forthcoming), TRS, WES
Variant Interpretation for Cancer Consortium (VICC)	cancervariants.org	international	Ca	24,366 evidence items	genetic and experimental evidence	centralized	public	Beacon V1, Service Registry / Info, VA (forthcoming), VRS

GA4GH Driver Projects are external genomic data initiatives that have committed to both contributing to the development of genomic data sharing standards as well as piloting their use in real world practice. Abbreviations: RD, rare disease; Ca, cancer; CT, complex traits; Bio, basic biology.

## Box 1 GA4GH Work Stream focus areas

The GA4GH Work Streams are the key production teams of the organization. Each tackles a specific area in the data life cycle, as described below (URLs listed in the web resources).

(1) **Data use & researcher identities**: Develops ontologies and data models to streamline global access to datasets generated in any country^9,10^
(2) **Genomic knowledge standards**: Develops specifications and data models for exchanging genomic variant observations and knowledge^18^
(3) **Cloud**: Develops federated analysis approaches to support the statistical rigor needed to learn from large datasets
(4) **Data privacy & security**: Develops guidelines and recommendations to ensure identifiable genomic and phenotypic data remain appropriately secure without sacrificing their analytic potential
(5) **Regulatory & ethics**: Develops policies and recommendations for ensuring individual-level data are interoperable with existing norms and follow core ethical principles
(6) **Discovery**: Develops data models and APIs to make data findable, accessible, interoperable, and reusable (FAIR)
(7) **Clinical & phenotypic data capture & exchange**: Develops data models to ensure genomic data is most impactful through rich metadata collected in a standardized way
(8) **Large-scale genomics**: Develops APIs and file formats to ensure harmonized technological platforms can support large-scale computing

## Box 2 Examples of GA4GH alignment with existing standards

By aligning with existing standards, tools, and resources, GA4GH aims to minimize redundancy and the unnecessary proliferation of competing standards. We outline three specific examples that demonstrate GA4GH efforts to align with existing standards and standards development organizations.

**Pedigree specification**: The PED format is a well-known standard for exchanging pedigree information and is widely used in both research and clinical settings (see PLINK in web resources).^20^ However, PED only allows for the representation of basic parent-child relationships, and does not represent all of the data elements and relationships needed by the genomics community. Building upon this format, the GA4GH Pedigree Subgroup has mapped PED format data elements to the Pedigree data model, allowing adopters to transition to a more robust representation of family health history without data loss and enabling compatibility with pre-existing family health history tools.

**Phenopackets specification**: Phenopackets, a standard for case-level phenotypic data exchange, can be compared to a hierarchical structure of “slots” that can be populated with ontology terms and other data. In order to maximize utility of computational analyses, these slots are compatible with any pre-existing terminologies or ontologies, such as the Human Phenotype Ontology for human disease phenotypes, NCI Thesaurus for cancer, LOINC for laboratory results, and MONDO for diseases. The modular design of the standard also enables interoperability with complementary GA4GH deliverables, like Pedigree and the Variation Representation Specification (VRS), by integrating them within the structure of the phenopacket.

**Genomic variation**: The GA4GH Variation Representation Specification (VRS) and Variant Annotation (VA) framework were developed to address the diverse methods used to access reference genome sequence and genomic annotation (e.g., genes, variation, regulatory regions, expression). Associated metadata can often be unstructured. VRS and VA aim to enable the provision, sharing, and computational representation of genomic variation information in a way that is unambiguous and semantically rigorous. These specifications are developed with bidirectional feedback with the standards of the health level 7 (HL7) clinical genomics working group, which supports the reporting of clinical genomic test results and related information with electronic health records (EHRs). Alignment between these specifications is a critical step toward supporting data exchange and system interoperability across the clinical-translational-research spectrum.

## Box 3 Major barriers hindering secondary use of clinically acquired data

Here we outline some of the major challenges to achieving the broad goal of responsible sharing of genomic and related health data. This includes setting up the infrastructure to support the flow of data from clinical practice into research, as well as establishing data-access and accountability mechanisms that are appropriate to research settings. These need to be consistent with the legal frameworks of the healthcare setting, and respectful of the rights of the individual data donor including their privacy, the security of their data, and their autonomy with regard to research participation.

1. Inconsistency and lack of version control in data-generating pipelines
2. Lack of dataset interoperability due to disparate data models and terminologies
3. Inadequate infrastructure for ingesting and storing data
4. Difficulty or lack of resources for enabling access to data
5. Insufficient consent for data sharing and lack of resources to support the consent process
6. Data privacy and security issues, as well as real and perceived regulatory issues
7. Challenges to ensuring patients understand how their data are used and have sufficient autonomy around data sharing participation
8. Differences in priorities, experiences, and trust levels concerning data sharing between different population groups and stakeholders
9. Lack of incentives in the clinical care system for prioritizing data sharing and research
10. Lack of data-sharing mandates

## Footnotes

**WEB RESOURCES**
All of Us Research Program, https://allofus.nih.gov
Australian Genomics, https://www.australiangenomics.org.au
Authentication & Authorisation Infrastructure (AAI) specification, https://github.com/ga4gh/data-security/blob/master/AAI/AAIConnectProfile.md
Autism Sharing Initiative, https://www.autismsharinginitiative.org/
Beacon Project, https://beacon-project.io/
Beacon Project API protocol, https://app.swaggerhub.com/apis/ELIXIR-Finland/ga-4_gh_beacon_api_specification/1.0.0-rc1
BRCA Exchange, https://brcaexchange.org/
Broad Data Use Oversight System (DUOS), https://duos.broadinstitute.org/dataset_catalog
Broad’s FireCloud - Data Library, https://portal.firecloud.org/#library
CanDIG, https://www.distributedgenomics.ca/
ClinGen, https://www.clinicalgenome.org/
COVID-19 data portal, https://www.covid19dataportal.org/
CRAM file format, https://samtools.github.io/hts-specs/CRAMv3.pdf
Data Use Ontology (DUO), https://raw.githubusercontent.com/EBISPOT/DUO/master/duo.owl
DNA.Land, https://dna.land/
DUO on Ontobee, http://obofoundry.org/ontology/duo.html
DUO on the Ontology Lookup Service, https://www.ebi.ac.uk/ols/ontologies/duo/terms?iri=http://purl.obolibrary.org/obo/DUO_0000001
ELIXIR, https://elixir-europe.org/
ENA browser, https://www.ebi.ac.uk/ena/browser/home
EpiShare, https://epishare-project.org/
EUCANCan, https://eucancan.com/
European Genome-Phenome Archive, https://ega-archive.org/
European Joint Programme on Rare Disease (EJP RD), https://www.ejprarediseases.org/
GA4GH, https://www.ga4gh.org/
GA4GH Clinical & Phenotypic Data Capture & Exchange, https://ga4gh-cp.github.io/
GA4GH Cloud Security and Privacy Policy, https://docs.google.com/document/d/1cBTwtetnsvO2vU3HVwLTLaC9H_ya-4MjZUa_g_xzOBg/edit
GA4GH Cloud Work Stream, https://ga4gh-cloud.github.io/
GA4GH Data Connect documentation, https://github.com/ga4gh-discovery/data-connect/blob/master/SPEC.md
GA4GH Data Connect specification, https://github.com/ga4gh-discovery/data-connect
GA4GH Data Privacy & Security, https://github.com/ga4gh/data-security
GA4GH Data Repository Service (DRS) API, https://github.com/ga4gh/data-repository-service-schemas
GA4GH Data Security Infrastructure Policy (DSIP), https://github.com/ga4gh/data-security/blob/master/DSIP/DSIP_v4.0.md
GA4GH Data Use & Researcher Identities, https://ga4gh-duri.github.io/
GA4GH Discovery Service Info, https://github.com/ga4gh-discovery/ga4gh-service-info
GA4GH Discovery Work Stream, https://ga4gh-discovery.github.io/
GA4GH file encryption standard, https://samtools.github.io/hts-specs/crypt4gh.pdf
GA4GH Genomic Knowledge Standards, https://ga4gh-gks.github.io/
GA4GH Large Scale Genomics Work Stream, https://github.com/ga4gh/large-scale-genomics-wiki/wiki
GA4GH Machine-Readable Consent Guidance (MRCG), https://www.ga4gh.org/wp-content/uploads/Machine-readable-Consent-Guidance_6JUL2020-1.pdf
GA4GH minimum dataset for family health history, https://docs.google.com/document/d/1UAtSLBEQ_7ePRLvDPRpoFpiXnl6VQEJXL2eQByEmfGY/edit?usp%20=%20sharing
GA4GH OpenAPI documentation, https://ga4gh.github.io/tool-registry-service-schemas/preview/develop/docs/index.html
GA4GH Passport specification, https://github.com/ga4gh-duri/ga4gh-duri.github.io/blob/master/researcher_ids/ga4gh_passport_v1.md
GA4GH Pedigree Standard, https://github.com/GA4GH-Pedigree-Standard/pedigree/blob/master/model.md
GA4GH Pedigree draft FIHR guide, https://github.com/GA4GH-Pedigree-Standard/pedigree-fhir-ig
GA4GH Pedigree Standard Family History Relations Ontology, https://github.com/GA4GH-Pedigree-Standard/family_history_terminology
GA4GH refget compliance suite, https://github.com/ga4gh/refget-compliance-suite
GA4GH Refget specification, https://github.com/ga4gh/large-scale-genomics-wiki/blob/master/refget.md
GA4GH regulatory and ethics toolkit, https://www.ga4gh.org/genomic-data-toolkit/regulatory-ethics-toolkit/
GA4GH RNAget API, https://ga4gh-rnaseq.github.io/schema/docs/index.html
GA4GH RNAget OpenAPI description, https://github.com/ga4gh-rnaseq/schema/blob/master/rnaget-openapi.yaml
GA4GH RNAget testing and compliance, https://github.com/ga4gh-rnaseq/schema/blob/master/testing/README.md
GA4GH service-info OpenAPI specification documentation, https://github.com/ga4gh-discovery/ga4gh-service-info/blob/develop/service-info.yaml
GA4GH Tool Registry (TRS) API, https://ga4gh.github.io/tool-registry-service-schemas/
Ga4GH TRS Swagger editor, https://editor.swagger.io/?url%20=%20https://raw.githubusercontent.com/ga4gh/tool-registry-schemas/develop/openapi/openapi.yam
Service Info Swagger Editor, https://editor.swagger.io/?url%20=%20https://raw.githubusercontent.com/ga4gh-discovery/ga4gh-service-info/develop/service-info.yaml
GA4GH Variant Annotation repository, https://github.com/ga4gh/va-spec
GA4GH Variation Representation Specification (VRS), https://vrs.ga4gh.org/en/stable/
GA4GH VRS example, https://vrs.ga4gh.org/en/stable/impl-guide/example.html#example
GA4GH VRS Python implementation, https://github.com/ga4gh/vrs-python/
GA4GH VRS relationship to existing standards, https://vrs.ga4gh.org/en/stable/appendices/relationships.html#relationships
GA4GH VRS repository, https://github.com/ga4gh/vrs
GA4GH Workflow Execution Service (WES) API, https://github.com/ga4gh/workflow-execution-service-schemas
GEnome Medical Alliance Japan (GEM Japan), https://www.amed.go.jp/en/aboutus/collaboration/ga4gh_gem_japan.html
Genomics England, https://www.genomicsengland.co.uk/
gnomAD, https://gnomad.broadinstitute.org/
Health Level Seven (HL7), http://www.hl7.org/
HL7 genomics reporting implementation guide, http://hl7.org/fhir/uv/genomics-reporting
Htsget, https://samtools.github.io/hts-specs/htsget.html
Human Cell Atlas, https://www.humancellatlas.org/
Human Heredity and Health in Africa (H3Africa), https://h3africa.org/
International Cancer Genome Consortium (ICGC) Accelerating Research in Genomic Oncology (ARGO), https://www.icgc-argo.org/
International COVID-19 Data Alliance (ICODA), https://icoda-research.org/
International Organization for Standardization (ISO), https://www.iso.org/home.html
Internet Engineering Task Force (IETF), https://www.ietf.org/
Matchmaker Exchange, https://www.matchmakerexchange.org/
Monarch Initiative, https://monarchinitiative.org/
National Cancer Institute Genomic Data Commons (NCI GDC), https://gdc.cancer.gov/
National Cancer Institute Cancer Research Data Commons (NCI CRDC), https://datascience.cancer.gov/data-commons
NIH policy for data management and sharing, https://grants.nih.gov/grants/guide/notice-files/NOT-OD-21-013.html
Open Biological and Biomedical Ontology Foundry (OBO), obofoun-dry.org/
OpenAPI description of refget v1.0.0, https://github.com/samtools/hts-specs/blob/master/pub/refget-openapi.yaml
Phenopackets, http://phenopackets.org/
Phenopackets GitHub repository, https://github.com/phenopackets/phenopacket-schema
PLINK, http://zzz.bwh.harvard.edu/plink/data.shtml
Public Health Alliance for Genomic Epidemiology (PHA4GE), https://pha4ge.org/
Refget documentation, https://samtools.github.io/hts-specs/refget.html
Refget summary public implementations, https://andrewyatz.github.io/refget-compliance/
Rare disease consent clauses, https://bmcmedethics.biomedcentral.com/articles/10.1186/s12910-019-0390-x/tables/3
SAM (Sequence Alignment/Map) file format, https://samtools.github.io/hts-specs/SAMtags.pdf
Service registry, https://github.com/ga4gh-discovery/ga4gh-service-registry
Swiss Personalized Health Network (SPHN), https://sphn.ch/
Variant call format (VCF), https://samtools.github.io/hts-specs/VCFv4.3.pdf
Trans-Omics for Precision Medicine (TOPMed), https://topmed.nhlbi.nih.gov/
The Task Execution Service (TES) API, https://github.com/ga4gh/task-execution-schemas
TRS human-readable Reference Documentation, https://ga4gh.github.io/tool-registry-service-schemas/
Variant Interpretation for Cancer Consortium (VICC), https://cancervariants.org/
Variant Annotation proposal, https://docs.google.com/document/d/1q8P1bjVyyslLcV8Gw_hXDc9JzOSuNbJyts-QDx1F17s/edit#heading=h.3e4s876j01gp
World Wide Web Consortium (W3C), https://www.w3.org/
