# Supplementary material for: GA4GH: International policies and standards for data sharing across genomic research and healthcare
Source: Cell Genom. Author manuscript; Available in PMC 2022 Jan 25. (PMC8774288; doi:10.1016/j.xgen.2021.100029)
Supplement: S1 [file EMS140859-supplement-S1.pdf]

Cell Genomics, Volume *1*

Supplemental information

GA4GH: International policies and standards for data sharing across genomic research and healthcare

Heidi L. Rehm, Angela J.H. Page, Lindsay Smith, Jeremy B. Adams, Gil Alterovitz, Lawrence J. Babb, Maxmillian P. Barkley, Michael Baudis, Michael J.S. Beauvais, Tim Beck, Jacques S. Beckmann, Sergi Beltran, David Bernick, Alexander Bernier, James K. Bonfield, Tiffany F. Boughtwood, Guillaume Bourque, Sarion R. Bowers, Anthony J. Brookes, Michael Brudno, Matthew H. Brush, David Bujold, Tony Burdett, Orion J. Buske, Moran N. Cabili, Daniel L. Cameron, Robert J. Carroll, Esmeralda Casas-Silva, Debyani Chakravarty, Bimal P. Chaudhari, Shu Hui Chen, J. Michael Cherry, Justina Chung, Melissa Cline, Hayley L. Clissold, Robert M. Cook-Deegan, Mélanie Courtot, Fiona Cunningham, Miro Cupak, Robert M. Davies, Danielle Denisko, Megan J. Doerr, Lena I. Dolman, Edward S. Dove, L. Jonathan Dursi, Stephanie O.M. Dyke, James A. Eddy, Karen Eilbeck, Kyle P. Ellrott, Susan Fairley, Khalid A. Fakhro, Helen V. Firth, Michael S. Fitzsimons, Marc Fiume, Paul Flicek, Ian M. Fore, Mallory A. Freeberg, Robert R. Freimuth, Lauren A. Fromont, Jonathan Fuerth, Clara L. Gaff, Weiniu Gan, Elena M. Ghanaim, David Glazer, Robert C. Green, Malachi Griffith, Obi L. Griffith, Robert L. Grossman, Tudor Groza, Jaime M. Guidry Auvil, Roderic Guigó, Dipayan Gupta, Melissa A. Haendel, Ada Hamosh, David P. Hansen, Reece K. Hart, Dean Mitchell Hartley, David Haussler, Rachele M. Hendricks-Sturrup, Calvin W.L. Ho, Ashley E. Hobb, Michael M. Hoffman, Oliver M. Hofmann, Petr Holub, Jacob Shujui Hsu, Jean-Pierre Hubaux, Sarah E. Hunt, Ammar Husami, Julius O. Jacobsen, Saumya S. Jamuar, Elizabeth L. Janes, Francis Jeanson, Aina Jené, Amber L. Johns, Yann Joly, Steven J.M. Jones, Alexander Kanitz, Kazuto Kato, Thomas M. Keane, Kristina Kekesi-Lafrance, Jerome Kelleher, Giselle Kerry, Seik-Soon Khor, Bartha M. Knoppers, Melissa A. Konopko, Kenjiro Kosaki, Martin Kuba, Jonathan Lawson, Rasko Leinonen, Stephanie Li, Michael F. Lin, Mikael Linden, Xianglin Liu, Isuru Udara Liyanage, Javier Lopez, Anneke M. Lucassen, Michael Lukowski, Alice L. Mann, John Marshall, Michele Mattioni, Alejandro Metke-Jimenez, Anna Middleton, Richard J. Milne, Fruzsina Molnár-Gábor, Nicola Mulder, Monica C. Munoz-Torres, Rishi Nag, Hidewaki Nakagawa, Jamal Nasir, Arcadi Navarro, Tristan H. Nelson, Ania Niewielska, Amy Nisselle, Jeffrey Niu, Tommi H. Nyrönen, Brian D. O'Connor, Sabine Oesterle, Soichi Ogishima, Vivian Ota Wang, Laura A.D. Paglione, Emilio Palumbo, Helen E. Parkinson, Anthony A. Philippakis, Angel D. Pizarro, Andreas Prlic, Jordi Rambla, Augusto Rendon, Renee A. Rider, Peter N. Robinson, Kurt W. Rodarmer, Laura Lyman Rodriguez, Alan F. Rubin, Manuel Rueda, Gregory A. Rushton, Rosalyn S. Ryan, Gary I. Saunders, Helen Schuilenburg, Torsten Schwede, Serena Scollen, Alexander Senf, Nathan C. Sheffield, Neerjah Skantharajah, Albert V. Smith, Heidi J. Sofia, Dylan Spalding, Amanda B. Spurdle, Zornitza Stark, Lincoln D. Stein, Makoto Suematsu, Patrick Tan, Jonathan A. Tedds, Alastair A. Thomson, Adrian Thorogood, Timothy L. Tickle, Katsushi

GA4GH Product Development & Approval Processes

** For the most recent and detailed version of the GA4GH product development and approval process, please refer to our website: [ga4gh.org/how-we-work/ga4gh-product-approval]*

Table of Contents:

- Introduction
- Product Proposal Process
- Development Process
- Product Approval Process
- Glossary

Introduction

This document outlines the processes by which the Global Alliance for Genomics and Health (GA4GH) develops and certifies technical standards and policy frameworks emerging from its work streams (WSs). GA4GH intentionally does not prescribe an in-depth and strict set of do's and don'ts. Each product comes with its own community and working practices. To enforce the same working practice across all products would be counterproductive and only increase the administrative burden of initiating new work or bringing external products into GA4GH. However, the organization does require that teams meet two specific milestones:

1) **Proposal**: before work on a new deliverable begins, the relevant Work Stream must present a proposal to the GA4GH Steering Committee, outlining the scope and expected impact of the new deliverable and the stakeholders that will be involved / affected.
2) **Approval**: before a new major version of a deliverable is released, it is submitted for approval and undergoes review by GA4GH committees as outlined below.

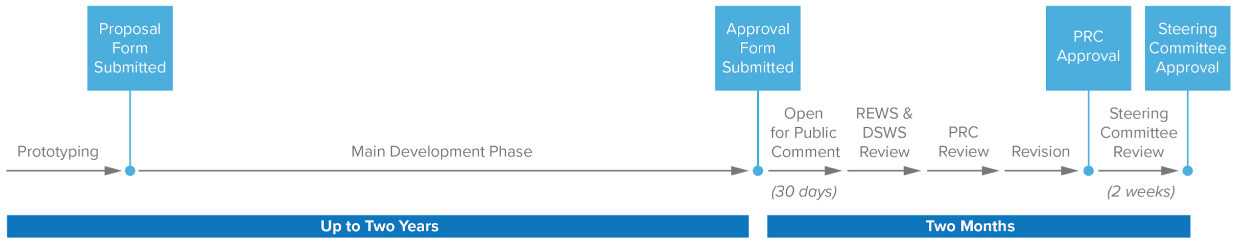

Product Proposal Process

Products are proposed and developed in response to the needs of the GA4GH Driver Projects (DPs)—real world genomic data initiatives that have committed to contributing to GA4GH standards development and implementation—or the wider genomics and health community. To maximize return on effort, WSLs prioritize development based on the number of requesting sources, leading to a limited number of deliverables with a greater adoption potential. WSs are

encouraged to include a broad landscape analysis in their initial prototyping activities; the specific details of this exercise is left to the discretion of the WSLs.

If WSLs decide to formally develop an initial prototype into a completed product, they work with the WS Manager (WSM) to create a Product Proposal slide deck to present to the GA4GH Steering Committee (SC). The groups that explicitly request a product are considered the "source projects" for that product. Each proposed product must be associated with at least two source projects. The product proposal deck and presentation at SC serves to notify other WSLs as well as DP Champions (DPCs) of the nature of the product being developed and to identify potential areas of overlap. In conjunction with the Product Proposal deck, the WSM also supports the team in completing the Data Security Product Proposal Questionnaire (reviewed by the Data Security Work Stream [DSWS] to ensure relevant security risks are considered) and the Regulatory and Ethics Questionnaire (reviewed by the Regulatory and Ethics Work Streams [REWS]). If significant issues are raised by anyone on SC or during the DSWS or REWS reviews, amendments may be made to the initial Product Proposal. Following these actions, the product is incorporated into the GA4GH Roadmap.

Development Process

GA4GH consists of eight WSs—two foundational and six technical. **Foundational Work Streams** provide guidance across GA4GH in the areas of regulation, ethics, and data security in genomics. **Technical Work Streams** develop GA4GH's technical standards and consist of leaders in their respective sub-disciplines from around the world. Technical WSs work to both discover and harden existing "working standards" and to create entirely new standards where needed. WS membership is voluntary and open to anyone. GA4GH welcomes additional interested individuals and organizations and encourages them to actively contribute across the organization by subscribing to emails, joining teleconferences, and attending face-to-face meetings.

GA4GH **Subgroups** are established within WSs to provide a focused space to develop and maintain specific deliverables. These groups also serve to guide future development activities and efforts to integrate the deliverable with other GA4GH products. Integration efforts also make use of Driver Projects, the Technical Alignment Subcommittee (TASC), and the Federated Analysis Systems Project (FASP) as ways of aligning and integrating deliverables.

Development work is carried out publicly and may use a system capable of tracking requests for changes, authorship, and history. In general, work such as program code, schemas, and documentation takes place in GitHub or Google Drive. Each WS and subgroup may have a unique approach to accepting changes, additions, and revisions to a standard, although most will recommend these be requested on GitHub trackers beginning with a pull request. See the GA4GH How We Work document for more details on the development process.

GA4GH specifications should meet the requirements of the source Driver Projects which requested them, or have had some feedback from the community in which it is intended to be

used. Development teams are encouraged to consider RFC 7282 from the Internet Engineering Task Force (IETF) to guide the decision making process.

Foundational Work Streams are contacted with Security, Regulatory, or Ethical concerns that arise during the development process.

Product Approval Process

When product leads and WSLs feel a GA4GH product is ready for a major version release, it is ***Submitted for Approval*** using the GA4GH Product Approval Submission Form. The GA4GH secretariat then notifies SC, as well as DSWS and REWS, which review the completed product using the DSWS and REWS review questionnaires. A Product Review Committee (PRC) is assembled, consisting of three members nominated by the submitter: (i) WSL from a different TWS, (ii) a member of a third Technical WS, and (iii) a representative from one of the product's source DPs.

The review committee may give a response of "Accept", "Reject", or "Changes Requested". All three members must agree unanimously for the committee to give a positive assessment. The committee should deliver their response one month after the specification is submitted.

If one of the three review bodies (REWS, DSWS, PRC) does not unanimously approve the product, requested upgrades will be communicated to the submitting WSMs. An upgraded product can be sent to the reviewers directly. This cycle can be repeated until the product passes the review body requirements.

Once all three bodies have approved the product, it is sent to the GA4GH SC for Approval. This must be done two weeks prior to the Steering Committee meeting at which it is to be assessed.

At the SC meeting, one WSL presents the product and the PRC may be called upon to explain the PRC decision. If SC votes unanimously to approve the product it becomes an **Approved GA4GH Deliverable**. If one or more SC members reject the product, the product may be required to make changes and then re-submit as described above. If the issues are minor enough. to allow the product to be re-considered in a single expedited review.

Approving New Versions

Minor and Patch updates to GA4GH products may take place without the Product Review Committee being re-convened. The approval of a new major version requires a new Product Approval submission, as described above.

Implementations

Each specification should have at least two associated implementations. These do not need to be officially maintained reference implementations. For a client-server model there should be at least two server deployments on separate implementation code bases interoperating successfully with two clients. These are viewed as best efforts and viewed as a way to ensure a robust product is developed. These implementations are not written or managed by WSs, but

rather by the Driver Projects and the community. Ideally, they are real world implementations of standards inside real-world environments that use real-world data. WSs facilitate interoperability testing between these implementations, which may lead to specification updates.

The Federated Analysis Systems Project (FASP) is a cross-WS initiative that aims to show that GA4GH APIs, when used in concert, can facilitate real-world, scientific use cases by conducting genomic analysis in the cloud. FASP aims to simulate how a researcher would search, access, and analyze genomic data within the GA4GH ecosystem via end-to-end test scenarios involving multiple Driver Projects. The FASP team also runs test scenarios against a wide variety of web service implementations, showing that common API specifications facilitate interoperability. This work involves the development of a comprehensive list of scientific use cases, as well as new web services for the test scenarios to run against. Learnings from FASP test scenarios are also fed back to the WSs and may lead to additional specification updates.

Retirement Process

WSLs may retire a product by submitting a request using the GA4GH Product Retirement Form. Any attribution or mention of GA4GH will be removed if appropriate. Products will be updated to point to their replacement if they have been superseded by a newer GA4GH Approved Product. GA4GH may choose to fork a product if the Secretariat deem a need to.

Glossary

- DP = Driver Project
- DPC = Driver Project Champion
- DSWS = Data Security Work Stream
- FASP = Federated Analysis Systems Project
- IETF = Internet Engineering Task Force
- PRC = Product Review Committee
- REWS = Regulatory & Ethics Work Stream
- SC = Steering Committee
- TASC = Technical Alignment Sub-Committee
- WS = Work Stream
- WSL = Work Stream Lead
- WSM = Work Stream Manager

Supplemental Information for *GA4GH: international policies and standards for data sharing across genomic research and healthcare*

Author Contributions

Conceptualization: A.A.P., A.B.S., A.D.P., A.F.R., A.Husami, A.H.W., A.K., A.M.L., A.Niewielska, A.R., A.V., B.H., B.J.W., B.M.K., B.P.C., D.B., D.Glazer, D.H., D.P.H., D.S., E.B., E.C.S., E.C.W., E.P., F.C., F.J., G.A., G.B., G.I.S., G.K., H.J.S., H.E.P., H.L.R., H.S., H.V.F., J.A.T., J.F., J.Nasir, J.O.J., J.P.H., J.P.W., K.Kato , K.N , K.P.E., K.T., K.W.R., L.F., L.L.T., M.Courtot , M.F., M.F.L., M.J.D., M.M., M.S., M.S.W., N.C.S., O.L.G., O.M.H., P.F., P.G., R.J.C., R.K.H., R.L., R.R.F, R.N., S.E.H., S.F., S.O.M.D, S.S., S.S.K., S.U., T.M.K., T.S., V.O.W., Z.S.

Data Curation: A.J., A.L.J., D.B., G.K., J.A.E., M.A.F., M.Courtot, M.M., N.M., S.Ogishima, T.Burdett.

Formal Analysis: A.Nisselle, D.F.V., D.L.C., J.M., R.J.M.

Funding Acquisition: A.A.T., A.E.H., A.T., B.J.W., B.M.K., D.B., E.B., G.B., G.I.S., H.E.P., H.L.R., H.N., J.A.T., M.A.H., N.C.S., P.F., P.G., R.R.F., R.K.H., S.J.M.J., S.V., T.S., W.G.

Investigation: A.H.W., C.Voisin, D.Glazer, E.C.W., E.M.G., J.M., J.P.H., J.P.W., K.Kosaki, K.P.E., L.D.S., L.J.D., M.Courtot, M.J.D., M.J.S.B., M.M.H., M.P.B., R.J.M.,R.K.H., R.L.G., S.Ogishima, T.Beck, T.Burdett., T.F.B., Z.S.

Methodology: A.B.S., A.D.P., A.F.R., A.H.W., A.Senf, C.Viner, D.B., D.F.V., D.Glazer, D.L.C., D.P.H., D.S., E.P., G.K., H.L.C., J.A.E., J.B.A., J.K., J.L., J.M., J.O.J., J.P.W., J.Z., K.Kosaki, K.T., K.W.R., L.J.B., M.Courtot, M.F.L., M.H.B., M.N.C., M.R., M.S., P.H., R.J.C., R.K.H., S.O.M.D, S.S.K., S.V., T.H.N., V.O.W., Z.S.

Project Administration: A.A.T., A.D.Y., A.E.H., A.J.H.P., A.K., A.L.J., A.L.M., A.Niewielska, A.R., A.Smith, A.T., B.D.O., B.J.W., B.M.K., D.P.H., E.M.G., G.B., G.I.S., H.E.P., H.N., J.A.T., J.C., K.A.E., K.A.F., L.I.D., L.F., M.A.F., M.C.M.T., M.Courtot, M.J.D., M.S., M.S.F., N.S., O.J.B., O.M.H., P.F., P.G., R.K.H., R.N., R.V.G., S.O.M.D, S.U., W.G.

Resources: A.A.P., A.A.T., A.Smith, B.P.C., C.Viner, D.M.H., H.N., D.P.H., H.V.F., J.A.T., J.O.J., M.S., N.C.S., S.H.C., S.V., T.M.K.

Software: A.A.P., A.D.P., A.H.W., A.K., A.M.J., A.Niewielska, A.P., A.Senf, B.D.O., B.H., B.P.W., C.K.Y., D.B., D.Glazer, D.Gupta, D.M.H., D.S., E.L.J., E.P., G.A.R., I.U.L., J.A.E., J.B.A., J.F., J.K., J.K.B., J.M., J.O.J., J.P.H., J.R., J.Z., K.P.E., K.W.R., K.Z., L.D.S., M.Courtot, M.Cupak, M.F., M.F.L., M.M., M.P.B., M.R., N.C.S., O.J.B., P.H., R.G., R.L., R.K.H., R.L.G., R.M.D., R.V.G., S.B., S.Ogishima, S.U., S.V., T.Beck, T.H.N., X.L.

Supervision: A.A.P., A.K., A.M., A.R., A.Smith, B.D.O., B.H., B.M.K., C.K.Y., D.B., D.C., D.D., D.Glazer, D.H., E.B., F.C., G.B., G.I.S., H.L.R., H.N., J.A.T., J.B.A., J.P.H., K.Kato, K.N., M.A.F., M.Courtot, M.F., M.F.L., M.J.S.B., M.M., M.S., N.C.S., O.J.B., O.L.G., O.M.H., P.F., P.G., R.R.F., R.G., R.J.M., R.K.H., R.M.C.D., R.N., R.V.G., S.B., S.E.H., S.F., S.J.M.J., S.O.M.D, S.R.B., S.U., T.Burdett., T.F.B., T.H.N., T.M.K

Validation: A.Hamosh, A.K., A.Niewielska, A.Smith, B.P.W., C.Voisin, C.Y., E.C.W., E.M.G., J.S.B., J.Z., K.K.L., L.J.D., L.J.Z., M.C.M.T., M.Courtot, M.P.B., P.H., R.K.H., R.S.R., S.Ogishima, S.U., S.V., T.F.B.

Visualization: D.B., H.L.C., K.W.R., L.J.Z., M.C.M.T., M.Courtot, S.B., S.L. .

Writing - Review & Editing: A.A.P., A.A.T., A.B., A.B.S., A.D.P., A.D.Y., A.E.H., A.F.R., A.Hamosh, A.Husami, A.J., A.J.H.P., A.K., A.L.J., A.L.M., A.M., A.M.J., A.M.L., A.Navarro, A.Niewielska, A.Nisselle, A.P., A.R., A.Senf, A.Smith, A.T., A.H.W., B.D.O., B.H., B.J.W., B.M.K., B.P.C., B.P.W., C.H., C.K.Y., C.Viner, C.Voisin, C.Y., D.B., D.C., D.F.V., D.Glazer, D.H., D.L.C., D.M.H., D.S., E.B., E.C.S., E.C.W., E.D., E.L.J., E.P., F.C., F.M.G., G.A., G.B., G.I.S., H.J.S., H.L.R., H.N., D.P.H., H.S., H.V.F., I.M.F., I.U.L., D.D., D.Gupta, J.A.E., J.A.T., J.B.A., J.C., J.K., J.K.B., J.L., J.M., J.Nasir, J.Niu, J.O.J., J.P.H., J.P.W., J.R., J.S.B., J.S.H., J.Z., K.A.E., K.A.F., K.K.L., K.Kato, K.Kosaki, K.N., K.P.E., K.T., K.Z., L.A.D.P., L.D.S., L.I.D., L.J.B., L.J.D., L.J.Z., L.L.T., L.S., M.A.F., M.A.K., M.Baudis, M.Brudno, M.C.M.T., M.Courtot, M.Cupak, M.F., M.F.L., M.G., M.H.B., M.J.D., M.J.S.B., M.L., M.M., M.M.H., M.N.C., M.P.B., M.R., M.S., M.S.C., M.S.W., N.C.S., N.M., N.S., O.J.B., O.L.G., O.M.H., P.F., P.G., P.H., P.N.R., P.T., R.A.R., R.C.G., R.R.F., R.G., R.J.C., R.J.M., R.L., R.L.G., R.M.C.D., R.M.D., R.M.H.S., R.N., R.S.R., R.V.G., S.B., S.E.H., S.E.W., S.F., S.H.C., S.J.M.J., S.L., S.Oesterle, S.Ogishima, S.O.M.D., S.R.B., S.S., S.S.J., S.S.K., S.U., S.V., T.B., T.F.B., T.H.N., T.L.T., T.M.K., T.N., T.S., V.O.W., W.G., X.L., Y.J., Z.S.

Writing - Original Draft: A.J.H.P., E.B., H.L.R., J.V., L.S., P.G.
