# Supplementary material for: GA4GH: International policies and standards for data sharing across genomic research and healthcare
Source: Cell Genom. Author manuscript; Available in PMC 2022 Jan 25. (PMC8774288; doi:10.1016/j.xgen.2021.100029)
Supplement: S2 [file EMS140859-supplement-S2.pdf]

GA4GH: international policies and standards for data sharing across genomic research and healthcare

Heidi L. Rehm[1,2], Angela J.H. Page[1,3], Lindsay Smith[4,3], Jeremy B. Adams[4,3], Gil Alterovitz[5], Lawrence J. Babb[1], Maxmillian P. Barkley[6], Michael Baudis[7,8], Michael J.S. Beauvais[9,3], Tim Beck[10], Jacques S. Beckmann[11], Sergi Beltran[12,13,14], David Bernick[1], Alexander Bernier[9], James K. Bonfield[15], Tiffany F. Boughtwood[16,17], Guillaume Bourque[9,18], Sarion R. Bowers[15], Anthony J. Brookes[10], Michael Brudno[18,19,20,21,39], Matthew H. Brush[22], David Bujold[9,18,39], Tony Burdett[23], Orion J. Buske[24], Moran N. Cabili[1], Daniel L. Cameron[25,26], Robert J. Carroll[27], Esmeralda Casas-Silva[125], Debyani Chakravarty[30], Bimal P. Chaudhari[31,32], Shu Hui Chen[33], J. Michael Cherry[34], Justina Chung[4,3], Melissa Cline[35], Hayley L. Clissold[15], Robert M. Cook-Deegan[36], Mélanie Courtot[23], Fiona Cunningham[23], Miro Cupak[6], Robert M. Davies[15], Danielle Denisko[19], Megan J. Doerr[37], Lena I. Dolman[19], Edward S. Dove[38], L. Jonathan Dursi[20,39], Stephanie O.M. Dyke[9], James A. Eddy[37], Karen Eilbeck[40], Kyle P. Ellrott[22], Susan Fairley[23,3], Khalid A. Fakhro[41,42], Helen V. Firth[43,15], Michael S. Fitzsimons[44], Marc Fiume[6], Paul Flicek[23], Ian M. Fore[29], Mallory A. Freeberg[23], Robert R. Freimuth[45], Lauren A. Fromont[52], Jonathan Fuerth[6], Clara L. Gaff[16,17], Weiniu Gan[33], Elena M. Ghanaim[46], David Glazer[47], Robert C. Green[48,1,49], Malachi Griffith[50], Obi L. Griffith[50], Robert L. Grossman[44], Tudor Groza[51], Jaime M. Guidry Auvil[29], Roderic Guigó[52,13], Dipayan Gupta[23], Melissa A. Haendel[53], Ada Hamosh[54], David P. Hansen[83,16], Reece K. Hart[124,1,100], Dean Mitchell Hartley[55], David Haussler[35], Rachele M. Hendricks-Sturrup[56], Calvin W.L. Ho[57], Ashley E. Hobb[6], Michael M. Hoffman[19,20,21], Oliver M. Hofmann[26], Petr Holub[58,59], Jacob Shujui Hsu[60], Jean-Pierre Hubaux[61], Sarah E. Hunt[23], Ammar Husami[62], Julius O. Jacobsen[63], Saumya S. Jamuar[64,65], Elizabeth L. Janes[66,3], Francis Jeanson[126], Aina Jené[52], Amber L. Johns[67,68], Yann Joly[9], Steven J.M. Jones[69], Alexander Kanitz[70,8], Kazuto Kato[71], Thomas M. Keane[23,72], Kristina Kekesi-Lafrance[9,3], Jerome Kelleher[73], Giselle Kerry[23], Seik-Soon Khor[74,75], Bartha M. Knoppers[9], Melissa A. Konopko[76], Kenjiro Kosaki[77], Martin Kuba[59], Jonathan Lawson[1], Rasko Leinonen[23], Stephanie Li[1,3], Michael F. Lin[78], Mikael Linden[79,80], Xianglin Liu[66], Isuru Udara Liyanage[23], Javier Lopez[101], Anneke M. Lucassen[81], Michael Lukowski[44], Alice L. Mann[15,3], John Marshall[68], Michele Mattioni[82], Alejandro Metke-Jimenez[83], Anna Middleton[84,85], Richard J. Milne[84,85], Fruzsina Molnar-Gabor[86], Nicola Mulder[87], Monica C. Munoz-Torres[53], Rishi Nag[23], Hidewaki Nakagawa[88,89], Jamal Nasir[90], Arcadi Navarro[91,92,52,93], Tristan H. Nelson[94], Ania Niewielska[23], Amy Nisselle[95,17,26], Jeffrey Niu[20], Tommi H. Nyrönen[79,80], Brian D. O'Connor[1], Sabine Oesterle[8], Soichi Ogishima[96], Laura A.D. Paglione[97,98], Emilio Palumbo[52,13], Helen E. Parkinson[23], Anthony A. Philippakis[1], Angel D. Pizarro[99], Andreas Prlic[100], Jordi Rambla[52,13], Augusto Rendon[101], Renee A. Rider[46], Peter N. Robinson[102,103], Kurt W. Rodarmer[,104], Laura Lyman Rodriguez[105], Alan F. Rubin[25,26], Manuel

Rueda[52], Gregory A. Rushton[1], Rosalyn S. Ryan[106], Gary I. Saunders[76], Helen Schuilenburg[23], Torsten Schwede[70,8], Serena Scollen[76], Alexander Senf[107], Nathan C. Sheffield[108], Neerjah Skantharajah[4,3], Albert V. Smith[109], Heidi J. Sofia[46], Dylan Spalding[79,80], Amanda B. Spurdle[110], Zornitza Stark[16,17,26], Lincoln D. Stein[4,19], Makoto Suematsu[77], Patrick Tan[64,111,112], Jonathan A. Tedds[76], Alastair A. Thomson[33], Adrian Thorogood[9,113], Timothy L. Tickle[1], Katsushi Tokunaga[114,75], Juha Törnroos[79,80], David Torrents[116,92], Sean Upchurch[115], Alfonso Valencia[116,92], Roman Valls Guimera[26], Jessica Vamathevan[23], Susheel Varma[117,23], Danya F. Vears[95,17,26,118], Coby Viner[19,20], Craig Voisin[119], Alex H. Wagner[31,32], Susan E. Wallace[10], Brian P. Walsh[22], Vivian Ota Wang[29], Marc S. Williams[94], Eva C. Winkler[120], Barbara J. Wold[115], Grant M. Wood, J. Patrick Woolley[73], Chisato Yamasaki[71], Andrew D. Yates[23], Christina K. Yung[4,121], Lyndon J. Zass[87], Ksenia Zaytseva[9,122], Junjun Zhang[4], Peter Goodhand[4,3], Kathryn North[17,26], Ewan Birney[23,123].

Summary

Scientific Editor:	Orli Bahcall
Initial submission:	2/10/2021
Revision received:	4/16/2021
Accepted:	6/14/2021
Rounds of review:	2
Number of reviewers:	3

Referee reports, first round of review

Reviewer #1: This is an overall robust, well written, timely, balanced and informative summary of the need for international standards and the ongoing role of GA4GH in the endeavor of bringing together clinical and research genomics. The organization of the paper - listing different efforts in the context of different classes of 'genetic' disorders, is sensible from the perspective of genomics disease researchers. I have a general comment and some very minor suggested edits.

The overarching general comment concerns the audience and the exact pitch of the paper and it's primary audience. Ideally, the commentary would be read by members of both the genomics research and the clinical care community. The obstacles to improving the flow of information between the clinic and the researcher are in both arenas, with arguably, most major obstacles coming from the clinical management end. The current paper

primarily addresses the trajectory of the genomics community and while it nicely makes the point that the genomic standards are necessary for their vision of the future, it does not address the other required ingredients. In particular, it does not discuss what is needed from clinical management community, for the end-game to be realized. Perhaps the authors do not believe that there needs to be active engagement from the clinical management community in order to enable the data-sharing vision that they subscribe to - and that the creation of standards such as those promoted by GA4GH will ipso facto lead to a genomic clinical-research bridge. Or perhaps they do not think this question is within the scope of this perspective. Some address to this question should, however, be within scope and while the authors cannot be expected to write in any detail about clinical management - the perceived role of the clinical community in the progress towards the vision should be clear in the manuscript.

If in agreement, the authors could (i) add some more verbage about the obstacles of EHRs and structured vs unstructured data are treated and (ii) add a section to address the point of the balance of responsibility of the clinical-management community, versus the research community, in completing development, implementing and propagating the standards they are advocating.

Very minor edits:
1: The authors have used the term 'Rare Disease' to include but extend upon 'Mendelian Disease'. That is fine - but they should state that explicitly, rather than implicitly, as they do in their opening sentence.
2: This sentence is garbled and should be broken into a couple of more clear, new sentences. "For example, there is a continuum between rare and common disease, and rare disease and highly penetrant cancer susceptibility can both be diagnosed and managed following similar clinical genetic pathways."

Reviewer #2:

Page and colleagues present a perspective on the state of genome sequencing over the next 4 years and the need for standardization to support global cooperation/collaboration - the raison d'etre for GA4GH.

The abstract is written clearly a but the paper seems more of a mosaic and lacks coherence and organization making it difficult to recognize the great strengths of the GA4GH and opportunities for impact in the future. Also the paper seems to flip back and forth between research and clinical applications. It seems like the great opportunity being discussed is the ability to co-opt clinical sequencing to drive a research agenda and vice versa - to capture the research findings and translate them to the clinical community. The end result of this work could be a genomics enabled learning health system model, which could be more clearly described and why and how standards and tools that GA4GH has developed would enable this important goal. the recently released NHGRI strategic plan has this concept front and center - how can GA4GH enable it?

Specific comments:

1. The three components of the abstract could be the basis for how the paper is organized - a) the differences between healthcare genomics and research genomic and the need for standards and policies for each and why, b) Drivers of the growth of clinical and research sequencing, c) the need for cohorts of > 10M and how sections above can get us there, and d) the GA4GH policy agenda that gets us there

2. The COVID example is an interesting one. One the one had there are global efforts but on the other there are many of them and how are they aligned and interoperable - are they? what role has GA4GH policies and tools played to allow for this to happen. what lessons learned from the pandemic can we use to develop a strategy for the next 4-5 years?

3. The section on GA4GH can be shorted or referenced. This has been covered in prior publications.

4. In passing it is mentioned that 'Research Genomes are, by convention, open to other researchers'. This is a significant feature of the research

enterprise that it has established this 'convention' - has it really? if it has, how did this come about?

5 The four case studies are interesting but what is lost and really not mentioned is a) what will be the anticipated sequencing in the next 4 years to arrive at the 60 million number quoted in the abstract and b) what will enable them? The section later in the paper describing the enabling efforts of GA4GH seem to be disconnected from this and in my opinion should be combined. The description of the clinical activities in the four areas seems tangential to the main topic at hand. what will enable the use of these sequences globally and how do we scale standards achieve the ultimate utility of sequencing for both the research and clinical community.

6. In the section on healthcare vs research it seems that there needs to be a discussion on are the incentives for health system to invest in the infrastructure for harmonization

incentives for federation
incentives for standardization
incentives for health systems to invest in the architecture
incentives for participants to share their data

7. a key issue that needs to be addressed is how do we measure the impact of the policies and standards, over what time and at what cost. some of this is buried and mentioned in passing in the latter sections on rare disease, chronic disease, cancer and ID. mention is made of the value that GA4GH has brought but more quantitative metrics are desirable.

8. The section highlighting the GA4GH tools and standards in the four areas could be tabulated for more clarity and links provided for the bolded text (file formats, Phenopackets, etc)

9. Table 2 should be brought into the text - how to build cohort of significant enough size, diversity (external and internal), phenotypic depth, follow up and how might these be sustainable

10. in the conclusion the pandemic is mentioned - what lessons have we learned from COVID 19 both the good and the bad. did we achieve the ideal state for the vision of GA4GH - at what cost and is it sustainable?

Reviewer #3: Introduction.
Lots of strong statements; very few citations of published supporting evidence.
One or two substantiating examples of the "unprecedented opportunities for rapid advancement of biological research through the study of the genetic and molecular components of health and disease on a massive scale". I might argue that the biggest barrier is the lack of a clear value proposition either for healthcare systems, payors or "data donors". Sure, such research will generate academic research papers, but that's very different from solving societal problems.
The barriers statements would be better in a text box.
While it's true that covid data repositories have grown at an unprecedented rate, what tangible benefits have arisen that would not have occurred otherwise?
The UN Declaration of Human Rights is misquoted. It actually states "Everyone has the right freely to participate in the cultural life of the community, to enjoy the arts and to share in scientific advancement and its benefits."
The introduction is too one sided. There are good reasons why individual patient data should be siloed - privacy, security, individual right to decide, protected groups, protected data fields. The authors fail to mention these. Nor do they make a compelling argument when and why the benefits exceed the risks.
GA4GH

The goal of GA4GH is mentioned, but no published charter is cited. Why will the "developing utility of genomics in clinical practice…." Be "higher risk" without such a consortium?
This section lacks a single citation of supporting evidence. At present the document reads as hyperbole. If there is to be merit in this document, it must be grounded in practical examples.
Genomics in healthcare
It is not true that the process of sequencing a genome is the same in any setting. This is a gross misunderstanding. Clinical genomes have stringently validated methods for all steps that are benchmarked extensively for analytic and diagnostic performance and clinical utility. They include multiple quality control checks. They are overseen by regulatory agencies who ensure that all aspects - laboratory, personnel, equipment and processes - conform to such metrics.
This section lacks a single citation of supporting evidence.
Rare Disease
The US definition of rare disease should also be quoted. The authors mix single locus genetic diseases with risk factors. The distinctions between rare diseases and single locus genetic diseases and risk factors should be clearly presented. OMIM is an excellent reference source for delineating the latter two.
The disparate goals of genomic sequencing in suspected affected vs unaffected single locus genetic disease should be mentioned. The subjects of etiologic versus clinical diagnosis, screening vs diagnosis, traditional medicine versus precision/genomic medicine should be discussed.
One of the biggest opportunities for large genome x phenome datasets is in describing the natural history of the several thousand, ultra-rare, single locus genetic diseases that have been discovered in the last decade. Their extreme rarity has the consequence that individual investigator or site studies will never accrue sufficient subjects to address this. Understanding natural history is a prerequisite to rational drug design for these diseases.
Cancer
This section lacks a single citation of supporting evidence. Again, the concepts of screening vs diagnosis and of primary versus recurrent cancer detection are missed. The repertoire of genome-informed treatments for cancer is still quite limited.
Common complex disease.
This section doesn't mention the common variant - common disease hypothesis that underpins PRS, nor the failure of GWAS to account for most genetic variation in many common diseases. There are well known successes of the common variant - common disease hypothesis in terms of clinical utility, but they aren't mentioned here.
Challenges
References are needed to recent, excellent articles on the learning healthcare system.
Technical Advances
There is no discussion of electronic health records systems. Most such systems were designed for accurate billing. Intrinsically that design means that the quality of the phenotype data that is extracted from them is poor. Discussion of structured versus non-structured text and machine-readable versus non-readable content is needed. There seems to be a lack of knowledge of the driving economics of healthcare delivery and its relationship to ballooning costs of electronic health records.
GRIN is an example of a federated large scale genomic data sharing resource. DNAnexus has established global data sharing capabilities.
In developing standards it is critical to mention the pre-existing vocabularies, standards and protocols on which GA4GH is building and their current strengths and weaknesses. A box that describes these discretely would be very helpful.
As I read this, I am very struck that for every challenge mentioned, there is a sentence such as "GA4GH seeks to…." or "GA4GH must enable…." followed by a generic solution statement. How? Why? Cite an example.
Societal Challenges
A principal societal challenge is education and engagement of the public, healthcare providers and regulators. The education and engagement only work when there are specific use cases where the benefits of large scale genomic medicine are clear.
"We envision clinical and basic researchers collaborating seamlessly in the context of practising healthcare." What is the origin of a principle statement like this? This statement displays quite a bit of ignorance of the nitty gritty details of healthcare delivery. There are potent reasons why it takes a decade to train a physician to do no harm.
GA4GH Standards

There really needs to be a section on economics. Without a sustainable business model, predicated on a clearly delineated, real world value proposition, very little of what is proposed will occur. There are potent reasons for countries not wanting to share their data across borders, and for healthcare systems not to share data with their competitors.
Who accredits GA4GH standards? Who substantiates their claims that the standards are generally applicable or harmonized or flexible or interoperable or portable? In actuality, we have a plethora of different ontologies and models that are not interoperable and that have not been benchmarked and, often, were not rationally designed for use in mathematical modeling. There are another plethora of accredited standards bodies who are answerable to professional or governmental communities…what is GA4GH doing to make sure that it is fully following, for example College of American Pathologists or DHSS regulations and standards?
Page 10. A large number of undefined terms of art start to be used - phenopackes, beacons, passports…a table that defines these would be useful.
Page 12. "Human Cell Atlas, NHGRI's AnVIL, EMBL-EBI pipelines, Genomics England Research Environment"…please define and cite these. Are they good, bad or indifferent? What are their relative strengths and weaknesses? Why were they chosen?
Page 13. "H3Africa, Genomics England, Australian Genomics, ClinGen, VICC, BRCA Exchange, Monarch Initiative, ICGC-ARGO, EUCANCan, and CanDIG"….please define and cite these.

Author response to the first round of review

No.	Comment	Response
First Review		
1.01	This is an overall robust, well written, timely, balanced and informative summary of the need for international standards and the ongoing role of GA4GH in the endeavor of bringing together clinical and research genomics. The organization of the paper - listing different efforts in the context of different classes of 'genetic' disorders, is sensible from the perspective of genomics disease researchers. I have a general comment and some very minor suggested edits.<br><br>The overarching general comment concerns the audience and the exact pitch of the paper and it's primary audience. Ideally, the commentary would be read by members of both the genomics research and the clinical care community. The obstacles to improving the flow of information between the clinic and the researcher are in both arenas, with arguably, most major obstacles coming from the clinical management end. The current paper primarily addresses the trajectory of the genomics community and while it nicely makes the point that the genomic standards are necessary for their vision of the future, it does not address the other required ingredients. In particular, it does not discuss what is needed from clinical management community, for the end-game to be realized.<br><br>Perhaps the authors do not believe that there needs to be active engagement from the clinical management community in order to enable the data-sharing vision that they subscribe to - and that the creation of standards such as those promoted by GA4GH will ipso facto lead to a genomic clinical-research bridge.<br><br>Or perhaps they do not think this question is within the scope of this perspective. Some address to this question should, however, be within scope and while the authors cannot be expected to write in any detail about clinical management - the perceived role of the clinical community in the progress towards the vision should be clear in the manuscript.	Thank you for pointing out this gap in the paper. We agree with the need to engage the clinical community and recognize the barriers there. We have added more content on this topic throughout the paper as well as two dedicated sections in the Challenges to Secondary Use of Clinically Acquired Data section entitled “Clinical Data Standards” and “Incentivizing and Facilitating Data Sharing in Healthcare”.

	If in agreement, the authors could (i) add some more verbiage about the obstacles of EHRs and structured vs unstructured data are treated and (ii) add a section to address the point of the balance of responsibility of the clinical-management community, versus the research community, in completing development, implementing and propagating the standards they are advocating.	
1.02	The authors have used the term 'Rare Disease' to include but extend upon 'Mendelian Disease'. That is fine - but they should state that explicitly, rather than implicitly, as they do in their opening sentence.	We have now added a clarifying statement that follows the stated definition of rare disease. "There are some non-genetic forms of rare disease (e.g. infectious, auto-immune), though for the purpose of this section, we are focusing on those rare diseases with a suspected genetic etiology."
1.03	This sentence is garbled and should be broken into a couple of more clear, new sentences. "For example, there is a continuum between rare and common disease, and rare disease and highly penetrant cancer susceptibility can both be diagnosed and managed following similar clinical genetic pathways."	Split into two sentences
2	Page and colleagues present a perspective on the state of genome sequencing over the next 4 years and the need for standardization to support global cooperation/collaboration - the raison d'etre for GA4GH.<br><br>The abstract is written clearly but the paper seems more of a mosaic and lacks coherence and organization making it difficult to recognize the great strengths of the GA4GH and opportunities for impact in the future. Also the paper seems to flip back and forth between research and clinical applications. It seems like the great opportunity being discussed is the ability to co-opt clinical sequencing to drive a research agenda and vice versa - to capture the research findings and translate them to the clinical community. The end result of this work could be a genomics enabled learning health system model, which could be more clearly described and why and how standards and tools that GA4GH has developed would enable this important goal. The recently released NHGRI strategic plan has this concept front and center - how can GA4GH enable it?	We have reorganized the paper and added content, including references to learning healthcare systems, as well as more clearly articulated why and how standards and tools that GA4GH has or will develop would enable our goals.
2.01	The three components of the abstract could be the basis for how the paper is organized -<br><br>a. the differences between healthcare genomics and research genomic and the need for standards and policies for each and why,<br>b. Drivers of the growth of clinical and research sequencing,<br>c. the need for cohorts of > 10M and how sections above can get us there, and<br>d. the GA4GH policy agenda that gets us there	We have restructured the summary to bring in the core areas of work of GA4GH as well as focus on key goals, including an outline of the paper's organization.

2.02	The COVID example is an interesting one. On the one hand there are global efforts but on the other there are many of them and how are they aligned and interoperable - are they? what role has GA4GH policies and tools played to allow for this to happen. What lessons learned from the pandemic can we use to develop a strategy for the next 4-5 years?	Based on other reviewer comments, we have removed the focus on COVID-19 and instead focused on other examples of successful global data sharing projects.
2.03	The section on GA4GH can be shortened or referenced. This has been covered in prior publications.	Significantly cut verbiage from first section after Introduction ("The Global Alliance for Genomics and Health") and replaced with edited/pared content from later section on GA4GH ("GA4GH Standards Enabling Genomic Medicine")
2.04	In passing it is mentioned that 'Research Genomes are, by convention, open to other researchers'. This is a significant feature of the research enterprise that it has established this 'convention' - has it really? if it has, how did this come about?	Rephrased the sentence to read, "Research genomes' are, more typically, open to other researchers based on funding and publishing requirements…"
2.05	The four case studies are interesting but what is lost and really not mentioned is<br>a) what will be the anticipated sequencing in the next 4 years to arrive at the 60 million number quoted in the abstract and<br>b) what will enable them?<br><br>The section later in the paper describing the enabling efforts of GA4GH seem to be disconnected from this and in my opinion should be combined. The description of the clinical activities in the four areas seems tangential to the main topic at hand. what will enable the use of these sequences globally and how do we scale standards to achieve the ultimate utility of sequencing for both the research and clinical community.	We have removed reference to the 60 million number as we decided not to focus on predicting this metric in this paper. We have now combined the disease-focused sub-sections of the "GA4GH Standards Enabling Genomic Medicine" section with the disease-focused sub-sections of the "Genomics in Healthcare" section and substantively reworked multiple sections to address the use of sequencing data and the need for standards and how use of this data will benefit both research and clinical care.
2.06	In the section on healthcare vs research it seems that there needs to be a discussion on are the incentives for health system to invest in the infrastructure for harmonization<br>● incentives for federation<br>● incentives for standardization<br>● incentives for health systems to invest in the architecture<br>● incentives for participants to share their data	We have addressed this topic in a new section entitled "Incentivizing and Facilitating Data Sharing in Healthcare".

2.07	A key issue that needs to be addressed is **how do we measure the impact of the policies and standards, over what time and at what cost**. Some of this is buried and mentioned in passing in the latter sections on rare disease, chronic disease, cancer and ID. Mention is made of the value that GA4GH has brought but more quantitative metrics are desirable.	We added a section called “Measuring success” as well as an example from Australian Genomics’ Acute Care pilot study which has shown that implementing GA4GH standards has directly led to a reduction in the time it takes for clinicians to complete an e-test order form.
2.08	The section highlighting the GA4GH tools and standards in the four areas could be tabulated for more clarity and links provided for the bolded text (file formats, Phenopackets, etc)	Added a table to the section titled, “The Global Alliance for Genomics and Health”, including links to and definitions of each standard.
2.09	Table 2 should be brought into the text - how to build cohort of significant enough size, diversity (external and internal), phenotypic depth, follow up and how might these be sustainable	Table 2 is a very large table and we don’t think physically moving it into the text is feasible. However, we have further focused on how GA4GH is enabling the ability to aggregate cohorts through common standards and methods of data exchange to enable sustainable approaches to solve this challenge.
2.10	in the conclusion the pandemic is mentioned - what lessons have we learned from COVID 19 both the good and the bad. did we achieve the ideal state for the vision of GA4GH - at what cost and is it sustainable?	We have removed focus on COVID-19 within the main body of the text based on reviewer comments and also revised the sentence in the conclusion to: “The recent response to the COVID-19 pandemic has shown us that the community is up to the task and supported by, organisations, governments, and individuals.”
3.01	Lots of strong statements; very few citations of published supporting evidence.	We have brought in many more citations throughout the manuscript.
3.02	One or two substantiating examples of the "unprecedented opportunities for rapid advancement of biological research through the study of the genetic and molecular components of health and disease on a massive scale". I might argue that the biggest barrier is the lack of a clear value proposition either for healthcare systems, payors or "data donors". Sure, such research will generate academic research papers, but that's very different from solving societal problems.	Added a sentence about translation of research discoveries into clinical care advances and added a reference to PMID: 24901184.
3.03	The barriers statements would be better in a text box.	Put list of barriers into a box

3.04	While it's true that covid data repositories have grown at an unprecedented rate, what tangible benefits have arisen that would not have occurred otherwise?	Based on multiple reviewer comments, we have removed the focus on COVID-19 and instead focused on other examples of successful global data sharing projects.
3.05	The UN Declaration of Human Rights is misquoted. It actually states "Everyone has the right freely to participate in the cultural life of the community, to enjoy the arts and to share in scientific advancement and its benefits."	We did not quote the Article verbatim - it was a paraphrase. Accurate verbatim quote now inputted.
3.06	The introduction is too one sided. There are good reasons why individual patient data should be siloed - privacy, security, individual right to decide, protected groups, protected data fields. The authors fail to mention these. Nor do they make a compelling argument when and why the benefits exceed the risks.	We have expanded on these issues adding: "including ensuring their privacy, security of their data and autonomy with regard to research participation" .To the following sentence "However, the challenge of setting up infrastructure to support the flow of data from clinical practice into the research setting is rivalled by the challenge of establishing data-access mechanisms that are appropriate to research applications, consistent with the legal frameworks of the healthcare setting, and respectful of the rights of the individual data donor including ensuring their privacy, security of their data and autonomy with regard to research participation.". We have also highlighted barriers by creating a box to better call out these issues and including an additional item "Ability to ensure that patients understand how their data is used and have sufficient autonomy around data sharing participation"
3.07	The goal of GA4GH is mentioned, but no published charter is cited. Why will "developing the utility of genomics in clinical practice…." be "higher risk" without such a consortium?	Replaced "it is clear" with "we believe"; Deleted "higher risk" and added reference to 2016 science paper as "published charter", as well as 2013 White Paper and 2017 BioRxiv paper
3.08	GA4GH section lacks a single citation of supporting evidence. At present the document reads as hyperbole. If there is to be merit in this document, it must be grounded in practical examples.	Added references to GA4GH papers from 2013 (Philippakis et al. 2013), 2016 (Page et al 2016), and 2017 (Birney et al. 2017) as well as the GA4GH website. We've also added 6 additional citations in the concluding paragraph of this section to provide examples.

3.09	It is not true that the process of sequencing a genome is the same in any setting. This is a gross misunderstanding. Clinical genomes have stringently validated methods for all steps that are benchmarked extensively for analytic and diagnostic performance and clinical utility. They include multiple quality control checks. They are overseen by regulatory agencies who ensure that all aspects - laboratory, personnel, equipment and processes - conform to such metrics.	Rephrased the sentence to read, "The process of sequencing a genome is essentially the same in any setting, but the scale and quality control of production as well as the regulation and dissemination of the resulting data can be quite different when used for healthcare versus research."
3.10	Genomics in Healthcare section lacks a single citation of supporting evidence.	Several references have been added to this section, including Marshall et al (2020), Vidgen et al (2020), and Tryka et al (2014), and Lin K-W (2013).
3.11	The US definition of rare disease should also be quoted. The authors mix single locus genetic diseases with risk factors. The distinctions between rare diseases and single locus genetic diseases and risk factors should be clearly presented. OMIM is an excellent reference source for delineating the latter two.	We have added the US definition and reworked this section to better clarify rare disease and its genetic components.
3.12	The disparate goals of genomic sequencing in suspected affected vs unaffected single locus genetic disease should be mentioned. The subjects of etiologic versus clinical diagnosis, screening vs diagnosis, traditional medicine versus precision/genomic medicine should be discussed.	We agree this is an important distinction but feel this topic is outside the scope of this article.
3.13	One of the biggest opportunities for large genome x phenome datasets is in describing the natural history of the several thousand, ultra-rare, single locus genetic diseases that have been discovered in the last decade. Their extreme rarity has the consequence that individual investigator or site studies will never accrue sufficient subjects to address this. Understanding natural history is a prerequisite to rational drug design for these diseases.	We agree with the reviewer and have added "(iii) define the natural histories of rare disease to predict disease progression and enable a foundation upon which to develop clinical trials;" as a key activity.
3.14	Cancer: This section lacks a single citation of supporting evidence. Again, the concepts of screening vs diagnosis and of primary versus recurrent cancer detection are missed. The repertoire of genome-informed treatments for cancer is still quite limited.	Added references to statements describing the role of genomic alterations in cancer, germline variants in cancer predisposition, time-scales for clinical decision making in cancers and consequences for applied genomic medicine, complications due to disease heterogeneity, the utility of genomic information in clinical decision making, and the need to analyze germline genomes for predisposition variants.

		We have also cited two select recent case studies demonstrating the real-world application of genomic data in cancers (Ricker et al. 2021; Moore et al. 2020). A much larger collection of studies is available for review in the *Molecular Case Studies* journal and elsewhere.<br><br>Additional text (with citations) was added to provide reference to the use of genomic information in the monitoring, diagnosis, prognosis, and therapeutic response prediction for cancer variants, with additional selected instances both in initial presentation and disease recurrence. Only a few were selected from the 7,667 curated studies currently available in the CIViC knowledgebase describing the role of 2,614 unique molecular alterations of potential use in clinical decision making for cancers (including evidence describing 462 distinct genome-informed treatments)
3.15	Common complex disease: This section doesn't mention the common variant - common disease hypothesis that underpins PRS, nor the failure of GWAS to account for most genetic variation in many common diseases. There are well known successes of the common variant - common disease hypothesis in terms of clinical utility, but they aren't mentioned here.	We have rewritten and added references to the common disease section including discussion of the common variant - common disease hypothesis.
3.16	Challenges: References are needed to recent, excellent articles on the learning healthcare system.	Added references to Britto et al. 2018, Serena et al. 2017, Levy et al. 2018, Zimmerman et al. 2016, and Williams et al. 2018 as well as PMID 21452449 and 24901184
3.17	Technical Advances: There is no discussion of electronic health records systems. Most such systems were designed for accurate billing. Intrinsically that design means that the quality of the phenotype data that is extracted from them is poor. Discussion of structured versus non-structured text and machine-readable versus non-readable content is needed. There seems to be a lack of knowledge of the driving economics of healthcare delivery and its relationship to ballooning costs of electronic health records.	We have addressed this topic in two new sections in the Challenges to Secondary Use of Clinically Acquired Data section entitled “Clinical Data Standards” and “Incentivizing and Facilitating Data Sharing in Healthcare”.

3.18	Technical Advances: GRIN is an example of a federated large scale genomic data sharing resource. DNAnexus has established global data sharing capabilities.	There are many consortium and commercial platforms like GRIN and DNAnexus that enable data sharing and our goal is not to reference them all. We decided to focus on national and internationally funded platforms in wide use and have edited the sentence to reference that.
3.19	Technical Advances: In developing standards it is critical to mention the pre-existing vocabularies, standards and protocols on which GA4GH is building and their current strengths and weaknesses. A box that describes these discretely would be very helpful.	Added discussion of alignment with existing standards, as well as general collaboration with external standards organizations, to the introduction.
3.20	As I read this, I am very struck that for every challenge mentioned, there is a sentence such as "GA4GH seeks to…." or "GA4GH must enable…." followed by a generic solution statement. How? Why? Cite an example.	Throughout the manuscript we have worked to provide more detail about the work we have done, why it is needed and what future plans we have.
3.21	Societal Challenges: A principal societal challenge is education and engagement of the public, healthcare providers and regulators. The education and engagement only work when there are specific use cases where the benefits of large scale genomic medicine are clear.	We agree this is an important point and have added an additional section as follows: "GA4GH has also launched the Genomics in Health Implementation Forum (GHIF; https://www.ga4gh.org/implementation), which is both a forum for Driver Projects and the broader community to share experience and best practices around implementing GA4GH standards, but also an opportunity to share broader experience in rolling out national and international data sharing activities. This forum and other areas of GA4GH engagement are critical to tackle the broader societal implementation issues including education and engagement of the public, healthcare providers and regulators in order to obtain the trust of the community."
3.22	Societal Challenges: "We envision clinical and basic researchers collaborating seamlessly in the context of practising healthcare." What is the origin of a principle statement like this? This statement displays quite a bit of ignorance of the nitty gritty details of healthcare delivery. There are potent reasons why it takes a decade to train a physician to do no harm.	We have edited, referenced and followed this statement with a clear and successful example of this happening today: "This has already been successfully modeled through the Clinical Genome Resource (ClinGen) where healthcare providers, clinical laboratory staff and researchers work together to develop standards for gene and variant curation, share underlying evidence and then

		apply that evidence through a consensus driven process to classify genes and variants which are then made freely accessible to the broader community (PMID: 30181607; PMID: 26014595).
3.23	GA4GH Standards: There really needs to be a section on economics. Without a sustainable business model, predicated on a clearly delineated, real world value proposition, very little of what is proposed will occur. There are potent reasons for countries not wanting to share their data across borders, and for healthcare systems not to share data with their competitors.	We respectfully disagree that a profitable economic business model is always necessary to enable sustainable data sharing and use of global standards. However, we do agree that demonstrating value of different types across our ecosystem is critical to our success. To that end, we have called out different motivating factors throughout the manuscript that help support the need for these standards and how the global community can work together to support them in a sustainable manner. We've also added an example of a clinical data sharing ecosystem that evolved without economic motivating factors which is ClinVar and the participation of the major commercial clinical labs in that effort (84% of data in ClinVar is from the top 20 submitters, 13 of which are commercial clinical labs https://www.ncbi.nlm.nih.gov/clinvar/docs/submitter_list/). This example and other concepts relevant to this topic are highlighted in a new section labeled " Incentivizing and Facilitating Data Sharing in Healthcare".
3.24	GA4GH Standards: Who accredits GA4GH standards? Who substantiates their claims that the standards are generally applicable or harmonized or flexible or interoperable or portable? In actuality, we have a plethora of different ontologies and models that are not interoperable and that have not been benchmarked and, often, were not rationally designed for use in mathematical modeling. There are another plethora of accredited standards bodies who are answerable to professional or governmental communities…what is GA4GH doing to make sure that it is fully following, for example College of American Pathologists or DHSS regulations and standards?	We have address this issue by adding two additional sentences to the beginning of the following paragraph: "Typically, GA4GH standards are not formally accredited by a national or international standards body and instead goes through an internal process of review by an independent panel of experts and voting by the steering committee of GA4GH, inspired by the Internet Engineering Task Force (IETF; https://www.ietf.org) and World Wide Web Consortium (W3C; https://www.w3.org) processes used in internet technologies. This allows us to be agile and quickly

		responsive to community needs and focus on lowering barriers to interoperability through the development and adoption of pragmatic standards by driver projects that commit to achieving meaningful exchange of data globally. However, there are occasions when certain standards may benefit from a more formal accreditation process, especially as we push more standards directly into healthcare usage. Therefore, to achieve greater international coordination and consistency of standards development, GA4GH proactively collaborates with other Standard Development Organizations (e.g., Health Level Seven (HL7), International Organization for Standardization (ISO) working in the genomics space. Without intentional coordination to keep respective products aligned, there is a risk of unnecessary proliferation of redundant standards, as well as the development of semantically- and syntactically-conflicting standards that will hamper large scale interoperability and introduce confusion within the adopter community.”
3.25	Page 10. A large number of undefined terms of art start to be used - phenopackes, beacons, passports…a table that defines these would be useful.	Added table of Product Roadmap with definitions of standards to section titled, “The Global Alliance for Genomics and Health” as first introduction to standards referenced later (pg. 10)
3.26	Page 12. "Human Cell Atlas, NHGRI's AnVIL, EMBL-EBI pipelines, Genomics England Research Environment"…please define and cite these. Are they good, bad or indifferent? What are their relative strengths and weaknesses? Why were they chosen?	Rewrote the sentence as several sentences, each describing how one of the named resources is providing cloud-based workflows for sharing, accessing, and analyzing large scale data. Provided citations for each.
3.27	Page 13. "H3Africa, Genomics England, Australian Genomics, ClinGen, VICC, BRCA Exchange, Monarch Initiative, ICGC-ARGO, EUCANCan, and CanDIG"….please define and cite these.	Added table of Driver Projects to section titled, “The Global Alliance for Genomics and Health” and referenced here.

Referee reports, second round of review

Reviewer #1: The positive: This revised version has addressed all the points that I raised and has in my view improved in these areas - in particular separately addressing the bridge to heath care.

The negative: Overall, however I am sorry to say that the article has new issues that have arisen, primarily, in response to the comment from other reviewers! It is a major re-write, hence the new comments. I recognize that this is an unusual situation and it will be up to the authors and editors to sort this out.

The new sections (other than 'the bridge to heath care') are largely centered upon the role, goals and strategic map for the consortium. I do agree with the other reviewer(s) who pointed out the benefits of being more explicit about the goals and operations of the program. However, rather than building the rationale for entire program, the authors have provided somewhat of a disorganized advertisement for GA4GH. They should have focused on a careful logical intellectual and practical argument as to why the program is needed. The new text is also more of a mosaic than before and the whole paper is a little 'choppy'.

There is some hubris: A small example to cite is the sentence 'The ultimate goal of GA4GH is to leverage the detailed knowledge of the genomics research community to advance healthcare and enrich biomedical research.' I would say this is already the goal of a large part of the international biomedical research community. The 'ultimate goal' of this group could properly be more modestly stated as '….to assist the ongoing activities of the genomics research community to advance healthcare and enrich biomedical research by advancing standards". This example should be contemplated throughout.

In the early part of the paper the authors begin to develop the discussion of the reason for the program but they miss the opportunity to build their story. Instead they jump from the paragraph beginning 'While efficient data storage and harmonization…' to 'GA4GH has partnered with 24 real-world genomic data initiatives ("Driver Projects") to ensure its standards are fit for purpose and driven by real-world needs. …" This is a big jump. Why not spend a paragraph or two explaining the gap in standards - THEN introduce the role of 'Driver Projects' to explain how such projects can use these standards and test out their practicability. This would be more logical flow.

As a result of the re-write, some of the latter paper is now redundant. E.g in the technical approaches section. That should be addressed.

Positive aspects of this version of the paper include most of the tables/figures/ text boxes. The new Box 3: Major Barriers Hindering Secondary Use of Clinically Acquired Data is very welcomed.
Exceptions to this enthusiasm for the tables/figures/ text boxes are:
(i) The new Matrix figure that is largely incomprehensible. It appears to be a word-association rather than a chart that can be referred to. I think a better figure would illustrate how standards evolve in some specific exemplary area.
(ii) Table 4. 'The need for cohorts of 10 million+ humans' now seems out of place. Some of this criticism could have been directed at this table in the first version - but with the re-write it seems even more out of place. The problem is two-fold. First, the logic flow of the paper is built on the premise that there will be a large increase in sample numbers. I think every one believes that. Second the contents of the table do not really fit any argument. Sections are redundant and issues like MatchMaker exchange make an appearance without much purpose. This table should be reduced dramatically and re-formed so it can be cited as 'Examples of how sample numbers will increase'. Or just delete.

Overall I still believe that the activities of this consortia comprise an important message to get out to the wider community. I liked more of the previous version than this one. I don't think it should be that hard to fix- some re-write of the earlier sections, less advertising, less redundancy and more stepwise building the arguments, particularly supporting the use of driver projects to test standards, would result in improvements.

Reviewer #2: The authors have done a great job in addressing my comments. Thank you.

Reviewer #3: The authors have responded to my concerns.

Author response to the second round of review

Second Review		
2.1	The new sections (other than 'the bridge to heath care') are largely centered upon the role, goals and strategic map for the consortium. I do agree with the other reviewer(s) who pointed out the benefits of being more explicit about the goals and operations of the program. However, rather than building the rationale for entire program, the authors have provided somewhat of a disorganized advertisement for GA4GH. They should have focused on a careful logical intellectual and practical argument as to why the program is needed. The new text is also more of a mosaic than before and the whole paper is a little 'choppy'.<br><br>There is some hubris: A small example to cite is the sentence 'The ultimate goal of GA4GH is to leverage the detailed knowledge of the genomics research community to advance healthcare and enrich biomedical research.' I would say this is already the goal of a large part of the international biomedical research community. The 'ultimate goal' of this group could properly be more modestly stated as '....to assist the ongoing activities of the genomics research community to advance healthcare and enrich biomedical research by advancing standards". This example should be contemplated throughout. In the early part of the paper the authors begin to develop the discussion of the reason for the program but they miss the opportunity to build their story. Instead they jump from the paragraph beginning 'While efficient data storage and harmonization…' to 'GA4GH has partnered with 24 real-world genomic data initiatives ("Driver Projects") to ensure its standards are fit for purpose and driven by real-world needs. …" This is a big jump. Why not spend a paragraph or two explaining the gap in standards - THEN introduce the role of 'Driver Projects' to explain how such projects can use these standards and test out their practicability. This would be more logical flow.	Thank you for the suggestions. We have incorporated the reframing language and added an additional paragraph to further explain the need for GA4GH. We also reorganized several sections to improve flow of the paper.

	Overall I still believe that the activities of this consortia comprise an important message to get out to the wider community. I liked more of the previous version than this one. I don't think it should be that hard to fix- some re-write of the earlier sections, less advertising, less redundancy and more stepwise building the arguments, particularly supporting the use of driver projects to test standards, would result in improvements.	
2.2	As a result of the re-write, some of the latter paper is now redundant. E.g in the technical approaches section. That should be addressed.	We have deleted the Technical Approaches section to address the redundancy
2.3	The new Matrix figure that is largely incomprehensible. It appears to be a word-association rather than a chart that can be referred to. I think a better figure would illustrate how standards evolve in some specific exemplary area.	We think the Matrix is important to understanding how GA4GH functions and figures can draw readers into a paper. However, to make this more useful, we have now included the full matrix with all mappings in the supplemental material and we note and link to that from the simplistic figure.
2.4	Table 4. 'The need for cohorts of 10 million+ humans' now seems out of place. Some of this criticism could have been directed at this table in the first version - but with the re-write it seems even more out of place. The problem is two-fold. First, the logic flow of the paper is built on the premise that there will be a large increase in sample numbers. I think every one believes that. Second the contents of the table do not really fit any argument. Sections are redundant and issues like MatchMaker exchange make an appearance without much purpose. This table should be reduced dramatically and re-formed so it can be cited as 'Examples of how sample numbers will increase'. Or just delete.	We have deleted Table 4
Editor Review		
Ed1	**Summary**: In the Abstract, please edit to more clearly highlight the top key messages and context.<br><br>Some notes:<br>● This perspective is to highlight the strategic vision for GA4GH, current progress and initiatives, and future plans<br>● And how this fits within landscape for research and healthcare – give some high level specifics<br>● And what are key deliverables and services will contribute<br>● And why this is a distinct and critically important initiative	We have restructured the summary to bring in the core areas of work of GA4GH as well as focus on key goals.

	• List key areas and sections presented in this perspective, with the context and relevance for GA4GH	
Ed2	**Introduction**: Not quite clear why the focus here is on COVID19 collaborations, although they are laudable. Perhaps you can streamline and bring a long term perspective on genomics, international collaborations, data sharing and standards.	We have removed the focus on COVID-19 and added several other examples of successful global data sharing projects. We've also highlighted in a box the major barriers to the secondary use of clinical acquired data and expanded to include lack of incentives per other reviewer feedback. We also added reference to learning healthcare systems per reviewer feedback.
Ed3	**The Global Alliance for Genomics and Health**: Can you introduce the strategic roadmap from the beginning, as the focus of this report. Introduce and also explain the context of the GA4GH structure, work streams, projects and deliverables, and how they are being released and implemented.	Introduced table of standards as they pertain to specific areas genomic medicine & research;<br>Moved high level description of Work Streams from appendix to the section "The Global Alliance for Genomics and Health";<br>Added table on GA4GH Driver Projects to this section.
Ed4	**GA4GH Standards Enabling Genomics Medicine**: This seems the most relevant of all sections – this could be included near the start in some form. It would also benefit from focusing and distilling main messages.	Moved preamble (with details of the suite of standards as a whole) into first section after introduction ("The Global Alliance for Genomics and Health"); moved disease-focused standards discussions into disease-focused challenges sections for greater continuity.
Ed5	**Additional edits and suggestions** were provided by the editor directly on the manuscript copy, including suggestions to focus and clarify the main messages and to streamline and reduce redundancy in the text to drive stronger impact.	All edits were either accepted or edited for clarity if meaning was incorrectly modified. Some sections were moved or revised to enhance clarity, or deleted if found to be redundant.
